# A Diverse Array of Fluvial Depositional Systems in Arabia Terra: Evidence for mid‐Noachian to Early Hesperian Rivers on Mars

**DOI:** 10.1029/2019JE005976

**Published:** 2019-07-22

**Authors:** Joel M. Davis, Sanjeev Gupta, Matthew Balme, Peter M. Grindrod, Peter Fawdon, Zachary I. Dickeson, Rebecca M.E. Williams

**Affiliations:** ^1^ Department of Earth Sciences Natural History Museum London UK; ^2^ Department of Earth Science and Engineering Imperial College London London UK; ^3^ School of Physical Sciences The Open University Buckinghamshire UK; ^4^ Planetary Science Institute Tucson AZ USA

**Keywords:** Mars, fluvial, climate, inverted channels

## Abstract

Branching to sinuous ridges systems, hundreds of kilometers in length and comprising layered strata, are present across much of Arabia Terra, Mars. These ridges are interpreted as depositional fluvial channels, now preserved as inverted topography. Here we use high‐resolution image and topographic data sets to investigate the morphology of these depositional systems and show key examples of their relationships to associated fluvial landforms. The inverted channel systems likely comprise indurated conglomerate, sandstone, and mudstone bodies, which form a multistory channel stratigraphy. The channel systems intersect local basins and indurated sedimentary mounds, which we interpret as paleolake deposits. Some inverted channels are located within erosional valley networks, which have regional and local catchments. Inverted channels are typically found in downslope sections of valley networks, sometimes at the margins of basins, and numerous different transition morphologies are observed. These relationships indicate a complex history of erosion and deposition, possibly controlled by changes in water or sediment flux, or base‐level variation. Other inverted channel systems have no clear preserved catchment, likely lost due to regional resurfacing of upland areas. Sediment may have been transported through Arabia Terra toward the dichotomy and stored in local and regional‐scale basins. Regional stratigraphic relations suggest these systems were active between the mid‐Noachian and early Hesperian. The morphology of these systems is supportive of an early Mars climate, which was characterized by prolonged precipitation and runoff.

## Key Points

- Fluvial depositional systems, 50–100 m in stratigraphic thickness, are common across Arabia Terra, Mars
- These deposits represent former rivers (at least 100–200 km long), floodplains, and lakes and are found filling fluvial valleys and basins
- These systems are mid‐Noachian to early Hesperian in age and represent significant periods of geologic time

## 1 Introduction

Numerous erosional valley networks dissect the Martian surface and are interpreted to have formed due to fluvial erosion, mostly around the Noachian/Hesperian boundary (~3.7 Ga) under arid to semiarid conditions (e.g., Carr, 1995, Craddock & Howard, 2002; Fassett & Head, 2008a; Hoke et al., 2011; Howard et al., 2005; Hynek et al., 2010; Irwin & Howard, 2002; Irwin et al., 2005; Ramirez & Craddock, 2018). However, models of the early Martian climate generally fail to predict an environment with temperatures above freezing, limiting the availability of liquid water (e.g., Forget et al., 2013; Wordsworth et al., 2013, 2015). Addressing this problem requires detailed studies of ancient Martian landscapes to place more robust geologic constraints on the nature of the early Mars climate (Hynek, 2016; Kite, 2019). Depositional systems can record long‐term environmental conditions and climate change and are the primary source of deep time paleoenvironmental data on Earth. On Mars, depositional systems have been used to infer early Martian conditions (e.g., Barnhart et al., 2009; Burr et al., 2010; Cardenas et al., 2017; Davis et al., 2018; DiBiase et al., 2013; Edgar et al., 2017; Fawdon et al., 2018; Goudge et al., 2016; Grindrod et al., 2017; Grotzinger et al., 2015; Kite et al., 2019; Rice et al., 2013; Williams et al., 2013; Wilson et al., 2016). However, most of these depositional systems likely record Hesperian environments; there is an urgent need to understand what Mars was like during the Noachian.

Davis et al. (2016) reported an extensive network of sinuous, branching ridges throughout Arabia Terra, an ancient region of Mars (middle to late Noachian ~3.9–3.7 Ga; Hynek & Di Achille, 2017; Tanaka et al., 2014), which borders the planetary dichotomy. Davis et al. (2016) interpreted the ridge systems as exhumed fluvial routing systems, remnants of fluvial channel deposits now inverted in the landscape. Such inverted deposits are sediment bodies formed by deposition in fluvial channel systems, which have been indurated and exhumed as ridges due to differential erosion (e.g., Burr et al., 2010; Maizels, 1987; Pain et al., 2007; Williams et al., 2009). The distribution of the Arabia Terra fluvial systems is consistent with formation by precipitation and runoff, and the deposits likely developed as part of a depositional fluvial system during the Noachian (Davis et al., 2016). Thus, extensive depositional systems in this region provide critical information on Martian paleoenvironments and by inference paleoclimate conditions during a time period for which little information is available.

Here, we build on the study of Davis et al. (2016), using high‐resolution image and topographic data sets to investigate the detailed morphology of the inverted fluvial deposits and show key examples of their stratigraphic and transitional relationships to associated fluvial landforms. In other locations across Mars, inverted channel deposits are sometimes associated with depositional basins, such as Aeolis Dorsa (e.g., Burr et al., 2010) and in the Eberswalde delta (e.g., Rice et al., 2013). The presence of ancient erosional valley networks to the south and throughout parts of Arabia Terra (Hynek et al., 2010) presents an opportunity to investigate the relationship of erosional valley networks that form part of the catchment to possible downstream fluvial depositional systems. We document how these relationships vary spatially throughout the region. Finally, we consider the formation environment for these fluvial systems and the implications for the regional Noachian climate that allowed such systems to develop.

## 2 Geology of Arabia Terra

Our study area comprises Arabia Terra, the most northerly region of the martian cratered highlands. The oldest exposed Noachian terrains probably comprise a mixture of volcanic deposits, impact breccias, and fluvial and aeolian sediments (e.g., Hynek & Di Achille, 2017). Much of Arabia Terra is covered by layered sedimentary rocks, the “etched units,” that are up to hundreds of meters thick, and which mantle, older Noachian terrain and have been extensively eroded (e.g., Edgett, 2005; Edgett & Malin, 2002; Fassett & Head, 2007; Hynek & Di Achille, 2017; Hynek & Phillips, 2001; Moore, 1990). The etched units may be reworked aeolian, dust, or pyroclastic deposits and are indicative of widespread regional resurfacing and denudation (e.g., Fassett & Head, 2007; Moore, 1990; Zabrusky et al., 2012). Limited fluvial dissection of the etched units suggests that the erosion was predominantly aeolian and mostly occurred at the Noachian/Hesperian boundary and early Hesperian (Fassett & Head, 2007; Zabrusky et al., 2012).

South of Arabia Terra, the Noachian erosional valley networks, which likely flowed north, become buried by the Hesperian‐aged units that make up Meridiani Planum (Hynek et al., 2010; Hynek & Di Achille, 2017; Hynek & Phillips, 2001). Although valley networks are generally absent in southwest Arabia Terra (Alemanno et al., 2018; Hynek et al., 2010), several large, regionally integrated erosional valley networks and intravalley basins traverse the central region of Arabia Terra (e.g., Naktong, Scamander Vallis), interpreted to have formed near the Noachian/Hesperian boundary (Fassett & Head, 2008a; Goudge et al., 2012; Irwin et al., 2005). Underlying the etched units is a network of branching and sinuous ridges, which comprise the remains of fluvial channel deposits (Chuang & Williams, 2018; Davis et al., 2016; Williams et al., 2018) and are predominantly found on middle to late Noachian age terrains (Hynek & Di Achille, 2017).

## 3 Data and Methods

**Figure 1:**
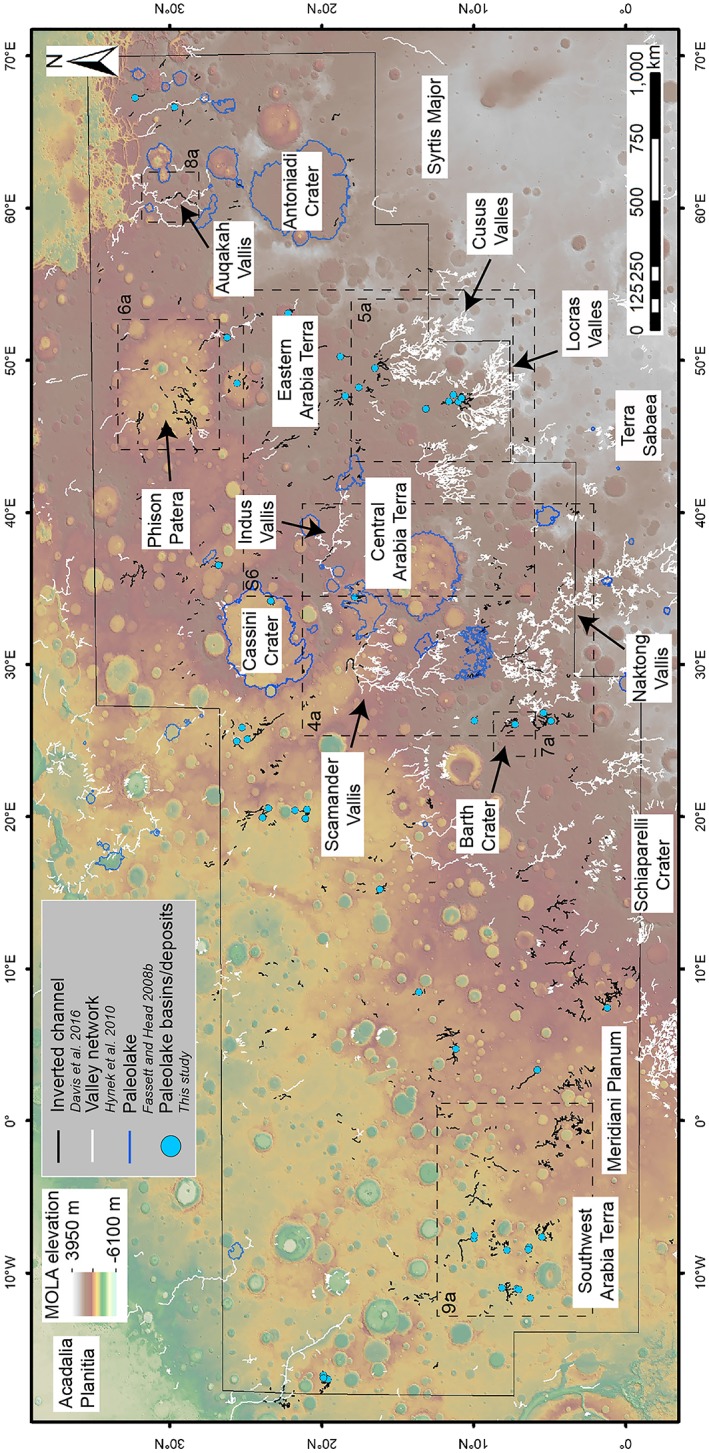
Distribution of branching and sinuous ridges interpreted as inverted channel systems (black lines) and associated landforms throughout Arabia Terra region on MOLA topographic map.

We investigated fluvial depositional systems across a broad study area in Arabia Terra (~0°N, 12°W to 35°N, 70°E; Figure 1) using a combination of Context Camera (CTX; 6 m/pixel; Malin et al., 2007) and High Resolution Imaging Science Experiment (HiRISE; 0.25 m/pixel; McEwen et al., 2007) image and topographic data sets. We produced HiRISE and CTX digital elevation models (DEMs; Table S1 in the supporting information) using the U.S. Geological Survey Integrated Software for Imagers and Spectrometers software and the BAE photogrammetric package SOCET SET (Kirk et al., 2008). The DEMs have a post spacing of 1 and 20 m/pixel for HiRISE and CTX, respectively, and where unavailable were supplemented by high‐resolution stereo camera (HRSC) DEMs (Jaumann et al., 2007) or Mars Orbiter Laser Altimeter (MOLA) topography (~450 m/pixel; Zuber et al., 1992).

We studied the relationship of the inverted channels to published data sets of valley networks and regional paleolake basins (Alemanno et al., 2018; Fassett & Head, 2008b; Hynek et al., 2010). New paleolakes were also identified in CTX data where basins, depressions, and sedimentary deposits intersect inverted fluvial channels. These paleolakes were mapped as point shapefiles, and their characteristics were recorded (i.e., basin or deposit and open or closed system). Finally, using these combined data, we examined the planview morphology, stratigraphy, and transitional relationships of the fluvial systems, to assess their formation environment.

## 4 Observations

Here, we summarize the structure of the observations section. In section 4.1, we describe the morphology of the various landforms throughout Arabia Terra and make provisional interpretations. We then describe specific examples of landforms that demonstrate key relationships and transitions throughout different regions of Arabia Terra: those landforms with regional catchments outside Arabia Terra (section 4.2), those with local catchments within Arabia Terra (section 4.3), and those with no clear catchment region (section 4.4).

### 4.1 Description of Landforms

#### 4.1.1 Morphology of Branching and Sinuous Ridges

**Figure 2:**
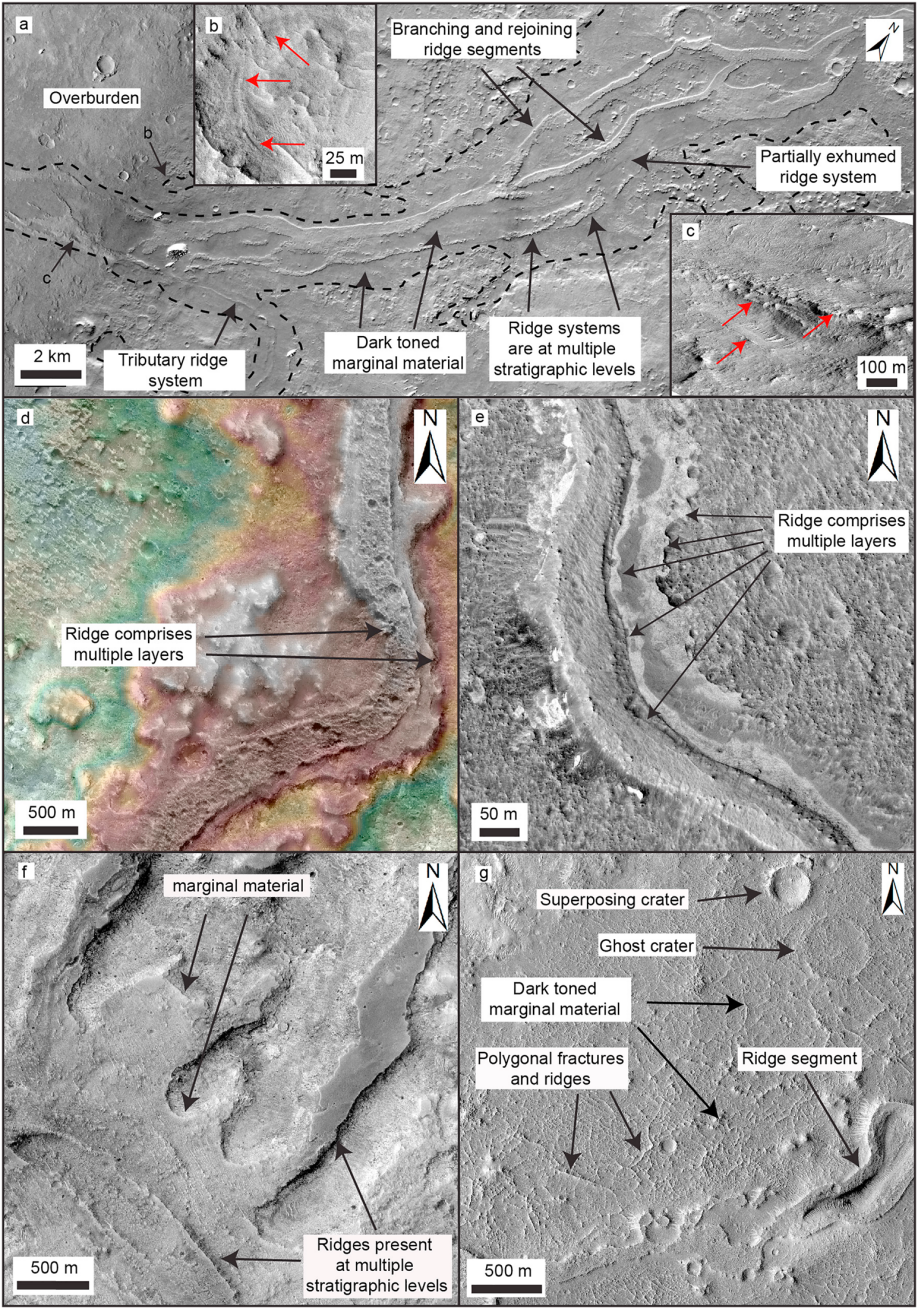
Examples of branching and sinuous ridge systems (inverted channel deposits) in Arabia Terra. (a) CTX mosaic of an inverted channel system (Aram Dorsum) exposed within an erosional window (dashed lines). Aram Dorsum comprises numerous, vertically stacked ridge systems, separated by dark‐toned marginal material. (b) HiRISE image showing subtle subhorizontal layering exposed in the deposits marginal to the ridges (red arrows). (c) Perspective HiRISE DEM image showing subhorizontal layering exposed in the ridge margins. (d) CTX DEM overlaid on a HiRISE image of a ridge system composed of multiple layers. (e) HiRISE image of ridge system composed of multiple layers with differing erosion resistance. (f) HiRISE image of ridges at different stratigraphic levels. (g) HiRISE image showing dark terrain marginal to ridge systems, which contains complex patterns of polygonal fractures and ridges.

Numerous branching and curvilinear ridge systems are exposed at the surface throughout Arabia Terra. (Figures 1, 2, and S1; e.g., Chuang & Williams, 2018; Davis et al., 2016; Williams et al., 2018; note the descriptions in this section are also applicable to those in section 4.1.4). These ridges are generally contiguous or semicontiguous for tens of kilometers, with the longest observed contiguous segment being ~200 km in length (six additional segments are greater than 100 km; Table S2). Ridges typically range in width from several tens of meters to 1–2 km and increase in width in the downslope direction, consistent with the scale and planimetric pattern of the valley networks. The height of the ridges ranges from several meters to around 50–100 m high (Figure S1). There is significant variability in the topographic profile of the ridges (both flat and rounded ridge crests are observed) and in their preservation style (see also Chuang & Williams, 2018).

The planview morphology varies between ridges systems: Most are branching (i.e., where multiple branches converge), and some are sinuous or linear (Figures 2, S1, and S2). Highly sinuous ridges (>1.5; defined as the ratio of the curvilinear length to Euclidean distance) are generally not observed. Some ridges comprise branches which diverge and rejoin (Figures 2, S1, and S2), although few systems that are entirely distributary in morphology are observed. Ridge segments can occur at the same elevation level as adjacent segments or form superposition relationships with older branches. Ridges are observed bound by and contiguous with erosional valley networks (section 4.1.4); however, terminal fan‐shaped depositional bodies associated with the ridges are not observed.

Meter‐ to decameter‐scale layering is present in some of the margins of the ridges (visible only in HiRISE images; Figures 2 and S1). We could not discern any structures within the layers such as inclined bedding. Given this, and that the layers generally run parallel to the long axis of the ridges, we assume the layers are near subhorizontal. The layers vary in brightness and sometimes form a “cliff and bench” erosional profile (Figures 2b and 2c), suggesting layers within the ridge have varying resistance to erosion. Some ridges are capped by a resistant, cliff‐forming layer, and multiple resistant, cliff‐forming layers are present in some ridge margins (e.g., Figures 2c–2e). These characteristics point to textural (e.g., grain size) and/or compositional variations within the ridges. Many ridges are vertically stacked (Figures 2a, 2f, and S2), demonstrating the presence of multilayered, temporally separated ridges systems (i.e., superposing ridges at different stratigraphic levels). Together, these characteristics are strong evidence that the ridges comprise exhumed fluvial deposits (inverted channel systems).

#### 4.1.2 Deposits Marginal to the Ridges

Stratigraphically adjacent to and underlying some of the ridges are rock outcrops that we term marginal materials (Figures 2 and S2). Generally, this material is dark in color (Figure 2a) and appears bright in THEMIS night data, although there are variations across different systems. The marginal material is generally smooth, except for meter‐ to decameter‐scale polygonal surface fractures and ridges, which are present in all HiRISE images we examined (Figures 2g and S2c). These polygons have junction angles of ~90–120° and appear best preserved adjacent to the ridge margins, although they sometimes extend into the ridges themselves (see Balme et al., 2016, for description of morphology at Aram Dorsum), indicating a possible association with the ridges. Subtle layering is sometimes exposed within impact craters in the marginal material (e.g., Figure 2b), suggesting that like the ridges, the marginal material is also sedimentary. The marginal material is not only present at the bases of the ridges but also occurs in between superposing ridge systems (e.g., Figures 2a, 2f, and S2c). Thus, like the ridges, the marginal material is also present at multiple stratigraphic levels.

Additionally, contained within the marginal material deposits are ghost craters, which are up to several hundred meters in diameter (Figure 2g) and have been infilled by overlying marginal material. The existence of ghost craters suggests that deposition of the marginal material was intermittent or episodic. In many examples, the marginal materials are poorly exposed, likely due to the extensive dust coverage across the Arabia Terra region (e.g., Poulet et al., 2007). Although the marginal material is difficult to interpret, its spatial association with and stratigraphic bracketing by the ridges suggests it may represent strata deposited during overbank or flood spillover events, which is consistent with a fluvial origin for the ridges.

#### 4.1.3 Basins and Indurated Sedimentary Mounds

**Figure 3:**
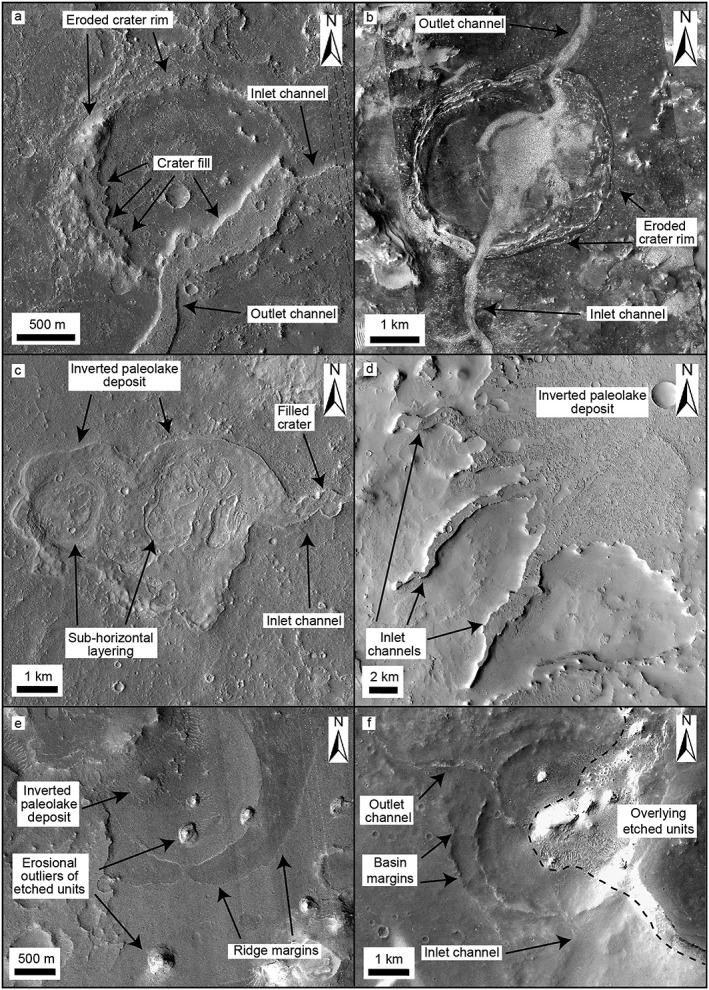
Examples of paleolake systems associated with inverted channel systems in Arabia Terra. (a) HiRISE image of infilled crater with an inlet and outlet inverted channel, forming an open‐basin paleolake. The rim has been extensively eroded and little ejecta deposits remain. Possible lacustrine deposits are found on the interior of the crater rim. (b) HiRISE and CTX image of open‐basin paleolake with inlet and outlet inverted channel. (c) CTX image of closed‐basin, inverted paleolake deposit. Subhorizontal layering visible in the top surface of the deposit is consistent with a sedimentary origin. (d) CTX mosaic of a possible inverted paleolake deposit in Cassini crater. (e) HiRISE image of inverted paleolake deposit associated with the Aram Dorsum inverted channel system in southwest Arabia Terra. The paleolake seems to have formed from a small spillover channel from the main channel. (f) CTX image showing open‐basin paleolake, with inlet and outlet inverted channels, that is overlain by etched units. Note that assessment between inlet and outlet channels was determined by topographic setting and channel configuration.

We identified numerous topographic basins and mound‐like indurated sedimentary deposits in association with the ridges (Figures 3 and S3 and Table S3). These features are distinct from the larger basins previously identified by Fassett and Head (2008b) and Goudge et al. (2012). The basins typically range in size from hundreds of meters to tens of kilometers across and were identified where ridge systems enter and exit a topographic low, such as an impact crater (Figures 3a and 3b). We interpret these basins as possible former closed or open lake systems (Fassett & Head, 2008b; Goudge et al., 2012).

Numerous mound‐like outcrops of indurated layered deposits with subcircular planforms are laterally contiguous with the ridges (Figures 3c–3f and S3a–S3c). Layering within the mounds that appears subhorizontal suggests they comprise sedimentary material (e.g., Figure 3c). The subcircular deposits are interpreted as the exhumed fill material of impact craters. If these locations were once impact craters, they have been extensively modified, with the crater rims and impact ejecta being almost entirely eroded away. Many of these deposits are present within larger crater basins (e.g., Cassini crater and Flammarion crater).

The close spatial relationship and textural similarity of these deposits to the ridge systems and the observation that they sometimes show ridges leading into and out of the subcircular deposits suggest that the formation of the ridge systems and the former crater infills is genetically related. Thus, the indurated sedimentary mounds are likely the site of paleolakes, which we term inverted paleolake deposits. Multiple indurated crater fill deposits without clear associated channels also exist in Arabia Terra; however, we chose not to include these examples as we cannot be certain they are paleolake deposits.

#### 4.1.4 Ridge Systems Within Erosional Valley Networks

**Figure 4:**
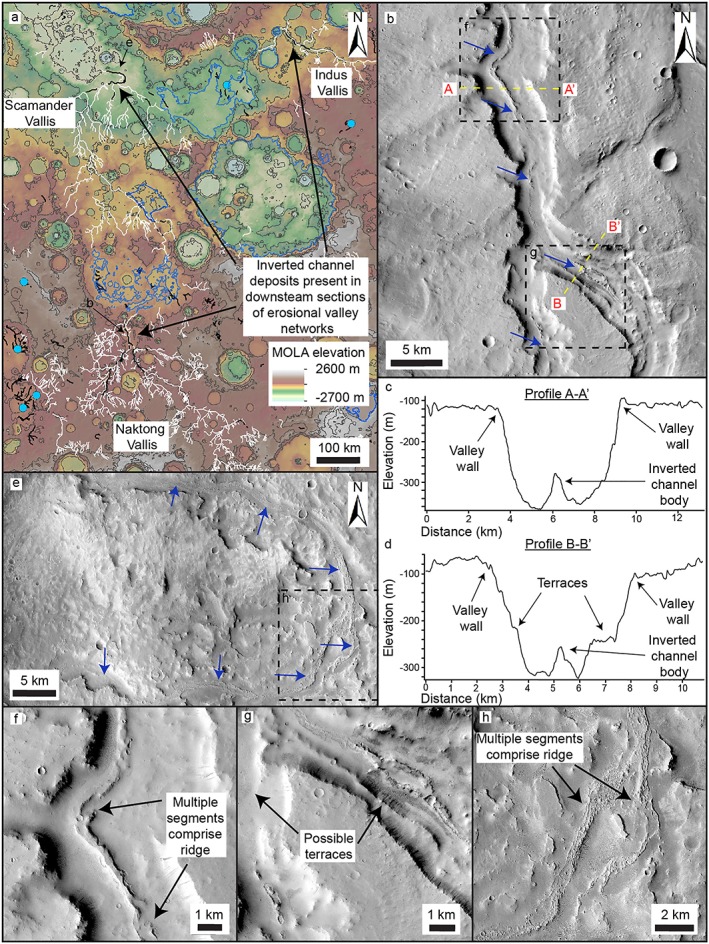
Valley‐inverted channel transition in the inferred Naktong/Scamander/Mamers Vallis system that traverses central Arabia Terra. (a) MOLA topographic map of central Arabia Terra. Erosional valley networks (white lines; Hynek et al., 2010) generally flow south‐north or southeast‐northwest and some contain inverted channel deposits (black lines). Also shown are large basins (Fassett & Head, 2008b; blue outlines) and paleolake basins and deposits associated with inverted channel deposits (blue dots). Contours shown at 500‐m intervals. (b) CTX mosaic of Naktong Vallis partially filled with inverted channel deposits (blue arrows) and erosional terraces. (c, d) CTX DEM profiles across Naktong Vallis. (e) CTX mosaic showing Scamander Vallis partially filled with inverted channel deposits. (f–h) CTX and HiRISE images showing inverted channel deposits in Naktong and Scamander Vallis.

**Figure 5:**
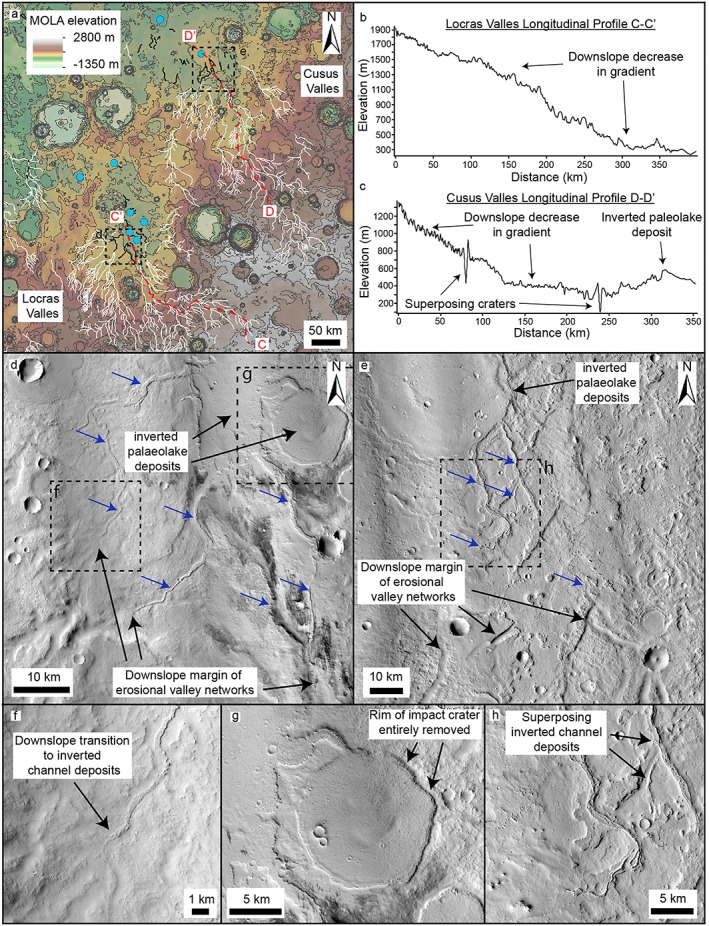
Downslope transitions at termini of Locras and Cusus Valles to nonconfined inverted channel deposits at the margins of basins. (a) MOLA topographic map, showing valley networks (white lines), which transition downslope into inverted channel (black lines) and paleolake deposits (blue dots). Contours shown at 200‐m intervals. (b and c) Topographic profiles from MOLA DEM showing decrease in gradient as valley networks approach the basin margins. (d) CTX mosaic showing basin at terminus of Locras Valles, which contains numerous inverted channel and paleolake deposits. (e) CTX mosaic showing inverted channel and paleolake deposits within a possible basin at terminus of Cusus Valles. (f) CTX mosaic showing nonconfined inverted channel deposits at the terminus of Locras Valles. (g) Inverted paleolake deposit within the basin at terminus of Locras Valles. (h) Superposing inverted channel deposits at terminus of Cusus Valles.

**Figure 6:**
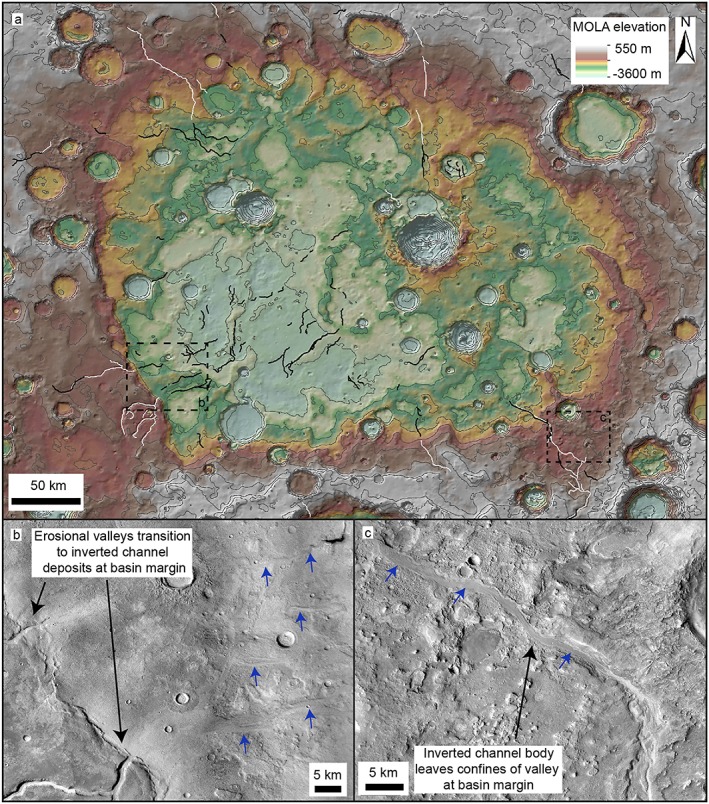
Valley‐inverted channels transitions at Phison Patera in northeast Arabia Terra. (a) MOLA topographic map showing Phison Patera, a ~400‐km closed‐basin system (absent from the Fassett & Head, 2008b, and Goudge et al., 2012, databases). Contours shown at 200‐m intervals. (b) CTX mosaic showing erosional valley networks transition to unconfined inverted channel systems at the southwest basin margins. (c) CTX mosaic showing valley‐bound inverted channel deposits transition into unconfined inverted channel deposits at basin margins.

**Figure 7:**
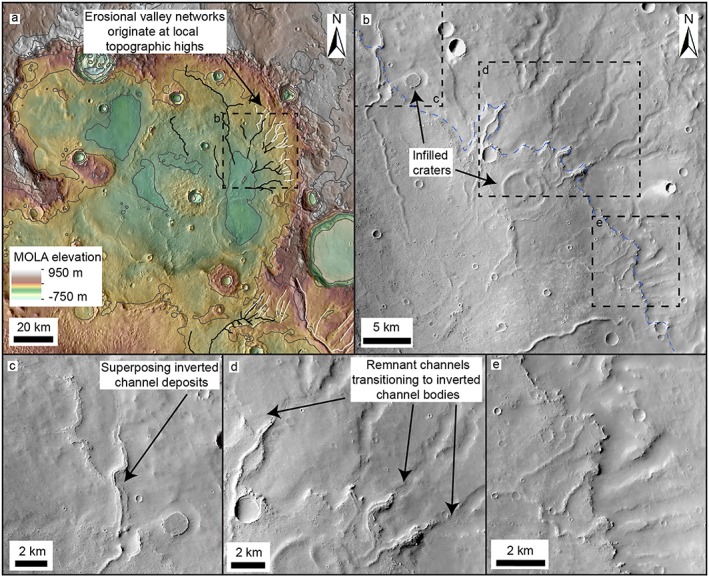
Valley‐inverted channel transitions within Barth crater in central Arabia Terra. (a) MOLA topography overlaid on a Thermal Emission Imaging System (THEMIS) infrared daytime basemap of Barth crater. Erosional valley networks (white lines) originate on the interior crater wall and transition into inverted channel systems (black lines). Contours shown at 200‐m intervals. (b) CTX mosaic showing valley‐inverted channel transition and associated deposits (blue dashed lines), possible alluvial or lacustrine material. (c) CTX mosaic showing superposing inverted channel deposits. (d) CTX mosaic showing remnant valley‐bound channel transitioning downslope into unconfined inverted channel deposits. (e) CTX mosaic showing scarp at the valley‐inverted channel transition boundary, suggesting the valley networks were once buried.

Evidence for the relationship between valley networks eroded into bedrock and the ridges comes from the observation of several valley networks that also contain exposures of ridges (e.g., Figures 4 and 5). These ridge‐forming strata are similar to those described earlier and interpreted as inverted channel deposits and occur at the base of erosional valley networks. These do not occupy the valleys for their entire length but occur in segments up to ~100 km long, predominantly in the lower reaches of the valleys. The erosional valleys are typically much wider (~2–5 km) than the interior ridge systems (~0.1–1 km). The ridges do not exceed the height of the valley walls; thus, the erosional valley networks are only partially filled. These valley‐confined ridges sometimes show downslope transition into unconfined ridge systems, usually at the margins of basins (e.g., Figures 5 and 6). Indurated sedimentary mounds are sometimes present in these basins contiguous with ridge systems (usually filling local accommodation space within the basin, but not the entire basin). Valley‐ridge transitions are also observed along the interior walls of crater basins (e.g., Figure 7).

### 4.2 Fluvial Systems With Regionally Integrated Catchments

In central and eastern Arabia Terra, the valley networks form large, regionally integrated catchments comprising multiple tributaries, >1,000 km in total length (Alemanno et al., 2018; Hynek et al., 2010) and are commonly associated with ridge systems. Here, we describe the relationship between the erosional valley networks and the ridge systems in three prominent examples: part of the Naktong/Scamander/Mamers (NSM) valley basin system (Fassett & Head, 2008b; Irwin et al., 2005), Locras Valles, and Cusus Valles.

#### 4.2.1 Naktong and Scamander Vallis

The NSM system forms a chain of semicontinuous erosional valley networks and intervalley basins which extends ~5,000 km from northwest Terra Sabaea, to the dichotomy, with an estimated watershed area of ~2.4 × 10^6^ km^2^ (Figures 1 and 4; Bouley et al., 2009; Fassett & Head, 2008b; Irwin et al., 2005). Several of the basins along the NSM system are breached by both inlet and outlet valleys and are interpreted as paleotopographic lows that were potentially occupied by open‐basin lakes (Fassett & Head, 2008b). Within several sections of Naktong, Scamander, and Indus Vallis (possibly connected to Scamander Vallis), segments of ridge systems are observed (Figure 4a). These segments extend for up to ~100 km and are ~0.2–1 km wide (Figures 4b and 4e–4h). Locally, examples of superposing ridges are sometimes observed. At least one pair of incisional terraces is present along some of the length of Naktong Vallis adjacent to the ridges (Figures 4b and 4d). Stacked ridge systems are observed in Naktong and Scamander Valles, although these generally do not reach the same elevation as the valley walls, or in the case of Naktong Vallis, the terraces (Figure 4). These ridge systems are generally concentrated in the downslope parts of the valley networks, in proximity to proposed paleolake basins (Fassett & Head, 2008b; Irwin et al., 2005; Figure 4).

#### 4.2.2 Locras and Cusus Valles

Two other erosional valley networks with regional integrated catchments, Locras and Cusus Valles, also show downslope transitions into ridge systems (Figure 5a). Both these networks drain several hundred kilometers north from northeast Terra Sabaea into Arabia Terra. The transition from valley network into ridge systems occurs at a break in slope where the fluvial systems enter basins (Figures 5a–5e). A striking observation is that preserved ridge systems are initially bounded within the confines of the valley network near the basin margins, before extending into the basin.

The ridge systems increase in width with distance into the basin, from ~100 m near the basin margins to over 1 km wide further into the basin (Figures 5d and 5e). Like the NSM system, multiple ridges are superimposed on top of one another (Figure 5h). The topographically confined basin that lies to the north of the outlet of Locras Valles is ~150 km across and ~300 m deep and likely formed a closed‐basin system (Figure 5a). The confines of the basin at the outlet of Cusus Valles are not well reflected in the current topography, except at its southern margins (Figure 5a). There is a possibility that it was ultimately connected to Indus Valles and thus the NSM system, potentially expanding its catchment area (Figure S6). While it is unclear if either of these basins was once entirely filled with sediment, the ridge systems are contiguous with smaller (km to tens of kilometers diameter), subrounded, indurated deposits, possible paleolake deposits (Figure 5g).

### 4.3 Fluvial Systems With Local Catchments

In several examples, fluvial systems with catchments that originate within Arabia Terra and are associated with ridge systems are observed. Here, we describe two examples of partially filled basins and one example of a ridge system truncated by an erosional valley network.

#### 4.3.1 Phison Patera

In northeast Arabia Terra, erosional valley networks enter a large (~10^5^ km^2^) basin, Phison Patera (Figure 6). Phison Patera is likely one or more highly modified impact craters and particularly notable because of its size and depth (>1 km in places), making it a large regional sediment sink. Erosional valley networks at the west and southeast basin margins are characterized by valley‐bound ridge systems, which show downslope transitions into ridge systems in an unconfined basin setting. Inverted channels here are ~0.1–1 km wide and are found up to 200 km away from the basin margins (Figures 6b and 6c). Several examples of highly sinuous ridge systems were identified within this basin which could be former meanders or preserved point bars; however, these are poorly preserved (Figure S2b).

#### 4.3.2 Barth Crater

Downslope directed negative‐ to positive‐relief transitions also occur locally within craters. Valley‐bound channels, which originate on the northeast interior wall of Barth crater, a ~100‐km crater in central Arabia Terra, transition downslope into unconfined, superposed ridge systems (Figure 7). The erosional valleys are ~0.5–1 km wide, and ridge systems are 100–500 m wide (Figures 7c–7e). The ridge systems are associated with layered material which has infilled the basin (Figure 7b). However, the ridge systems and this associated material are topographically higher than the upslope valley networks, suggesting the valley networks were once buried. It is unclear whether the basin is an open or closed system: The southern margins of Barth crater have been removed and stratigraphic relationships are obscured by overlying ridged plains material, which does not appear to be associated with the fluvial valleys or ridge systems.

#### 4.3.3 Auqakah Vallis

**Figure 8:**
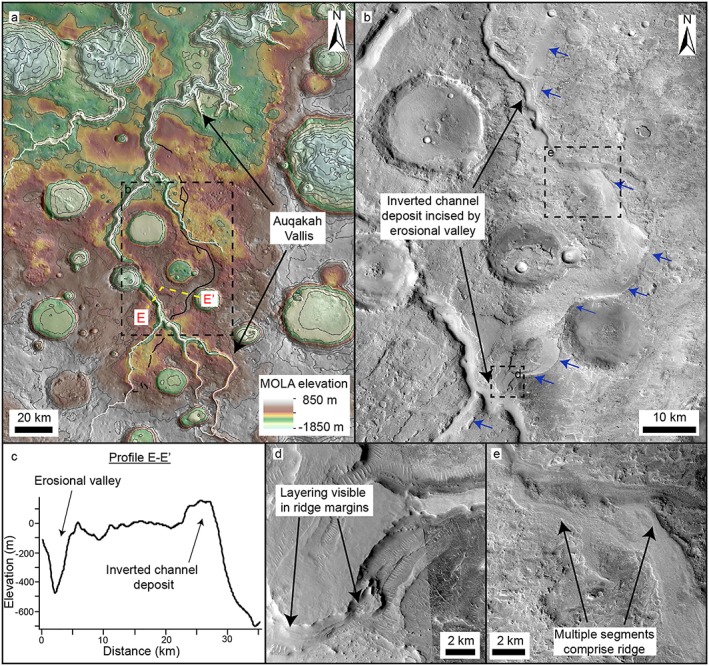
Inverted channel deposit incised by an erosional valley. (a) MOLA topography on a THEMIS infrared daytime basemap showing Auqakah Vallis in eastern Arabia Terra (contours shown at 200‐m intervals). (b) CTX mosaic showing an inverted channel deposit (blue arrows) which has been incised by Auqakah Vallis (c) Topographic profile E–E′ extracted from a HRSC DEM. The inverted channel deposit is ~100 m high. (d) CTX mosaic showing layering within the channel margins. (e) CTX image showing that the ridge comprises numerous channels or branches.

Auqakah Vallis is an erosional valley network which drains from north of Antoniadi basin for several hundred kilometers north toward the dichotomy scarp (Figure 1). At two separate locations, Auqakah Vallis is incised ~600 m through a ~1–2‐km‐wide, ~100‐m‐high ridge, oriented north‐south (Figure 8). The upper surface of the ridge suggests that the landform comprises multiple ridges, which have become laterally amalgamated (Figure 8e).

### 4.4 Ridge Systems Without a Clear Preserved Source Region

**Figure 9:**
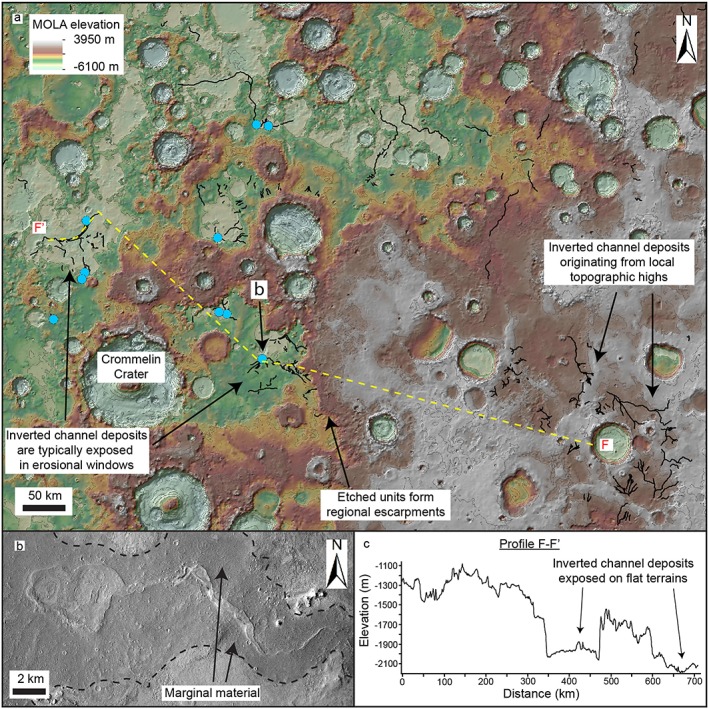
High density of inverted channel deposits in southwest Arabia Terra region. (a) MOLA topographic map showing inverted channel systems (black lines) and inverted paleolake deposits (blue dots) that have no clear preserved catchment. (b) Context Camera image of inverted channel system terminating in inverted paleolake deposit with corridors of dark marginal material which runs parallel to the channel. (c) Topographic profile F–F′ extracted from MOLA DEM. Inverted channel systems are typically found on flat terrain exposed in erosional windows.

For some ridge systems, no clear relationship to a preserved upland source catchment area is observed. This is particularly apparent in southwest Arabia Terra region, north of Meridiani Planum, where the largest concentration of ridge systems occurs (Figure 9). These ridge systems are some of the widest and most contiguous within the study area (multiple segments 1–2 km wide and up to 100–200 m long are present). Although the topography in this region is highly varied, the erosional windows that the ridge systems are exposed within are flat; end‐to‐end ridge‐top gradients are typically ~0.001–0.002 (measured from MOLA topography; Figure 9c). Sharp changes in the orientation of ridge segments suggest they may have been deflected around topographic barriers such as impact craters (Figure 9a). The ridge systems in this region intersect numerous crater basins and indurated mounds, possible paleolake systems. Dark corridors of marginal material run parallel to some ridges systems here, which we interpret as marginal material (Figure 9b).

These ridge systems do not preserve evidence of a clear source region and do not show a direct relationship to any erosional valley network. One possible source region are the upslope valley networks that are located to the south of and buried by Meridiani Planum (Figure S4). The valley networks here incise the same geological unit that many of the inverted channel systems in southwest Arabia Terra are found on (Nhc_1_; mid‐Noachian; Hynek & Di Achille, 2017; Figure S4); however, the stratigraphically younger Meridiani Planum units obscure the nature of these relationship. Several ridge systems also originate from local topographic highs in the southeast of the region (Figure 9a); however, this is unlikely to be the only source region, given the size of the area over which the ridges are found.

## 5 Discussion

### 5.1 Development of Aggradational Channel‐Belt and Flood Plain Deposits

We interpret the sinuous ridge systems as the preserved remnants of self‐formed alluvial channel deposits. The existence of multilayered, superposing ridges is strong evidence that the ridges probably do not comprise the fill of individual, isolated geomorphic channel threads. This is a highly unlikely occurrence in stratigraphic records as rivers inherently migrate through time. Instead, the characteristics of the ridge systems are best explained if they comprise the exhumed remains of channel‐belt and floodplain deposits, which aggraded vertically (e.g., Friend et al., 1979). We suggest that the ridge systems comprise a succession of ribbon‐form sandstone and conglomerate channels bodies and interstratified overbank deposits and that the material marginal to the inverted channels comprises fine‐grained overbank deposits dominated by mudstones and siltstones. This interpretation is supported by the sinuous, branching, and reconnecting planview morphology of the ridge systems, the cliff and bench erosional profile of the ridges, the superposition and horizontal amalgamation of multiple ridges, and the characteristics of the marginal material (layering, interstratifcation with the ridge systems) adjacent to the ridge systems.

By contrast, we do not interpret the ridges as eskers or glacial moraines as the ridges (1) conform to regional topography (whereas eskers do not necessarily; e.g., Butcher et al., 2017), (2) lack any associated glacial landforms, and (3) are often associated within fluvial valley networks (e.g., Hynek et al., 2010), which do not show signs of glacial modification. We also discount a lava flow interpretation due to (1) the layering within the ridge margins providing strong evidence for a sedimentary origin (e.g., Figures 2b, 2c, and 3c) and (2) a lack of associated volcanic structures. Our interpretation of the ridge systems as exhumed channel‐belt and floodplains deposits is consistent with interpretations of similar landforms on Earth (e.g., Utah; Hayden et al., 2019; Williams et al., 2009).

The induration of the channel deposits was likely due to channel armoring or the cementation of channel sediment (e.g., Burr et al., 2010; Pain et al., 2007; Williams et al., 2009). As part of a global study of large‐scale inverted channel deposits (including many in Arabia Terra), Williams et al. (2018) suggested that induration via cementation best explains their thermophysical properties in most cases. The cementation of fluvial sediment may also produce the consolidated rock necessary to explain the erosion history of the ridges (Williams et al., 2018), some of which have been exhumed from beneath ~1 km of overlying material (e.g., Zabrusky et al., 2012).

Aggrading alluvial systems composed of channel belts and floodplains can develop where fluvial systems confined in bedrock valleys enter topographic basins. Here, the loss of confinement at entry points and common decrease in gradients result in broad‐scale sediment deposition. The basins provide “accommodation space” for sediment accumulation. This interpretation is consistent with the settings where the inverted channels are observed in Arabia Terra: across low‐relief plains (Figures 1 and 9), emerging from erosional valley networks at basin margins (Figures 5 and 6) and in the lower reaches of erosional valley networks (Figures 4). There is little evidence for lateral channel migration in the form of scroll bar morphologies or well‐developed channel meanders. Channels likely aggraded vertically to create a fluvial succession at least ~50–100 m thick (as inferred from the ridge heights). Moreover, we might expect great thicknesses of the alluvial succession to be buried beneath the surface.

### 5.2 Incision and Filling of Erosional Valley Networks and Basins

**Figure 10:**
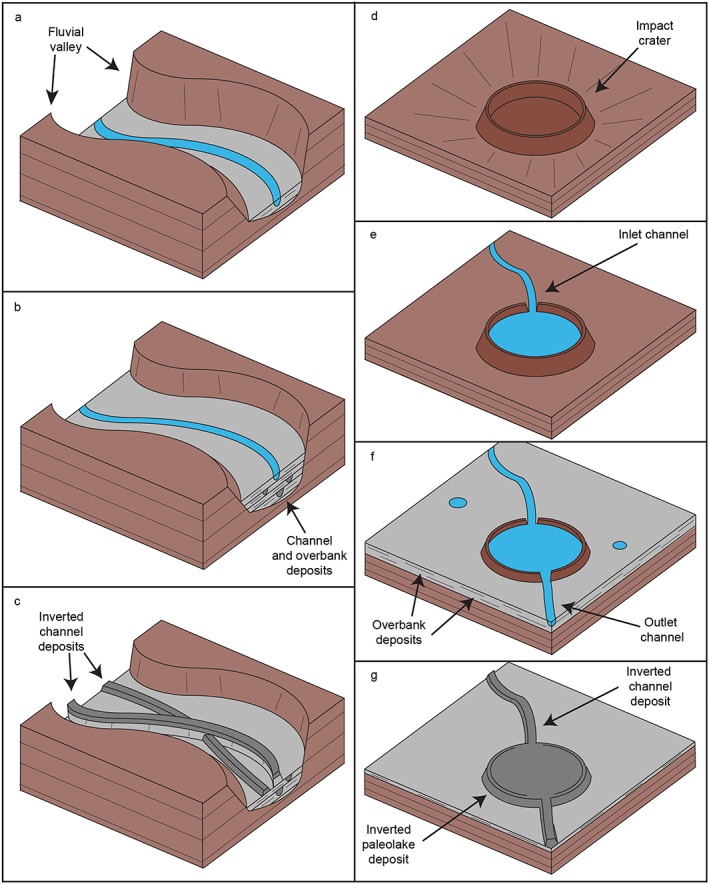
Diagram showing interpreted evolution of (a–c) partially filled valley networks and (d–g) inverted paleolake deposits. (a) Incision leads to formation of fluvial valley. (b) Possible changes in sediment or water flux, or a rise in base level causes the valley to become filled with channel‐belt deposits. (c) After fluvial activity ceases, erosion of the overbank material exhumes the channel deposits, forming inverted channel deposits within the valley. Parts a–c is adapted from Cardenas et al. (2017). (d) Impact crater forms. (e) Fluvial activity leads to erosion of crater and formation of a crater lake. (f) Overbank deposits and/or other sedimentary deposits buildup and lake breaches over crater rim, forming an outlet channel. (g) After fluvial processes cease, overbank material is eroded, exhuming indurated channel and lake sediment, which forms inverted channel and paleolake deposits.

The morphology of the erosional valley networks in central and eastern Arabia Terra is consistent with a history of initial bedrock valley incision followed by fluvial sediment aggradation within the valley. Fluvial downcutting occurred to form the ~100‐ to 250‐m‐deep valleys (possibly multiple downcutting events in the case of Naktong Vallis, which contains bedrock strath terraces), with later aggradation of channel‐belt deposits (Figure 10).

Previous studies suggest the Naktong and Scamander section of the NSM system formed during a proposed intense phase of fluvial erosion at the Noachian/Hesperian boundary (Fassett & Head, 2008b; Hoke et al., 2011; Irwin et al., 2005). The presence of channel‐fill in the downstream portion of these erosional valleys suggests a phase of deposition followed this period of erosion. These processes could plausibly be driven by changes in water or sediment supply, or by a rise in base level, as is seen in filled valley successions on Earth (e.g., Blum & Asnan, 2006; Singh et al., 2017). The formation of impact craters could have altered flow regimes or created local dams, which could have produced such effects. Additionally, the proximity of several possible regional paleolake basins to the filled sections of the NSM system (Figure 4; Fassett & Head, 2008b) suggests that these valley segments could have been subject to backwater effects as the water levels in basins fluctuated (Blum et al., 2013). The initial downcutting phase(s) may have increased the amount of coarse sediment, which was then available to form bank‐stable depositional channels (Cardenas et al., 2017; Dietrich & Smith, 1983). The formation of large impact ejecta blankets during the time that the fluvial systems was forming may have also provided a source of unconsolidated sediment.

Downslope transitions to unconfined inverted channel systems also occur where erosional valley networks enter basins (e.g., Locras, Cucus Valles, and Phison Patera; Figures 5 and 6). Unlike Naktong and Scamander Vallis, Locras and Cusus Valles do not appear to have been significantly backfilled, suggesting that the transitions here may have been driven by a reduction in slope leading to the formation of depositional channels and sediment aggradation, rather than water‐level fluctuations, or alternatively, this could be due to preservation bias. As the inverted channel deposits extend into the basin, they appear to have infilled local accommodation space (e.g., craters; Figure 5), forming paleolake systems. These characteristics suggest that the lake systems did not occupy the basin; the basin was instead probably occupied by alluvial systems, although smaller connected lakes may have existed within them. Similar relationships are observed along the interior walls of Barth crater, where valley‐bound channel deposits show transitions into unconfined inverted channel systems (Figure 7), possibly due to a reduction in slope. However, as the downslope inverted channel and associated marginal deposits are topographically higher than the upslope valley networks, the valley networks may have been buried during a period of lake‐level rise in the basin.

An unusual valley network‐inverted channel relationship is observed at Auqakah Vallis, where the ~100‐m‐thick inverted channel is incised by a ~600‐m‐deep erosional valley (Figure 8). This stratigraphic relationship suggests that the channel system has been indurated and exhumed, only to be later incised by fluvial activity, which may indicate a pause in fluvial activity. However, as the inverted channel system has been incised by Auqakah Vallis, fluvial activity later resumed, indicating that fluvial processes may have been episodic or intermittent in at least this region. The ~600‐m‐deep incision by the erosional valley could have been induced by a fall in base level, triggering a period of fluvial erosion.

### 5.3 Formation of Inverted Paleolake Deposits

One of the interesting results of our study is the identification of mound‐like stratified bodies that we interpret as paleolakes, based on their continuity with the inverted channel deposits. The sedimentary strata comprising these deposits could be (1) fluviolacustrine sedimentary infills of crater lakes that became indurated (e.g., Edgett, 2005); (2) secondary material which postdates the paleolake, such as cemented aeolian or dust deposits or volcanic ash, possibly associated with the emplacement of the etched units (e.g., Fassett & Head, 2007); or (3) a combination of the above (i.e., fluviolacustrine material overlain by nonfluviolacustrine material). Due to their lateral continuity with the inverted channel deposits, we suggest that the majority of the stratified mounds comprise indurated fluviolacustrine sediment (inverted paleolake deposits) and probably formed in a similar mechanism to the inverted channels (i.e., cementation; Pain et al., 2007; Williams et al., 2018).

As the inverted paleolake deposits were likely once impact craters, their rims and ejecta must have been removed by erosion. This probably occurred mostly prior to sediment deposition; it seems unlikely that crater fill would be preserved, while impact crater rims and ejecta were entirely removed. Many other impact craters elsewhere on Mars have had their rims and ejecta blankets similarly removed, consistent with fluvial erosion (Craddock & Howard, 2002; Craddock et al., 1997). Craters are sometimes visible that have been partially modified but still have their rims intact (e.g., Figures 3a and 3b). Given the context of the fluvial systems throughout Arabia Terra, fluvial erosion could be responsible for this modification of impact craters. Water and sediment were then deposited in the now modified impact craters, forming paleolakes (assuming the sediment in the paleolakes is lacustrine; Figure 10). The sequence of erosion followed by later sediment accumulation is consistent by the observations of other landforms in Arabia Terra (e.g., partially filled valleys and basins). We recognize that this mechanism is likely an oversimplification of a complex, possible multistage fluvial process.

### 5.4 Fluvial Sediment Transport in Arabia Terra

The preserved remnants of fluvial depositional sedimentary bodies in Arabia Terra reveal complex yet important spatially diverse sediment routing systems. Our observations show that water and sediment were sourced both regionally (e.g., Figures 4 and 5) and locally within Arabia Terra (e.g., Figures 6 and 7). In central and eastern Arabia Terra, some of the inverted channel systems show clear connections to highland valley networks (e.g., the NSM system, Locras and Cusus Valles). There is evidence that water and sediment was stored in local paleolakes and regional‐scale intervalley‐network basins, which may have acted as sediment sinks (e.g., Phison Patera, Locras and Cusus basins). From the inferred transport direction and the regional slope, which are generally south‐to‐north and southeast‐to‐northwest, it is plausible that the eventual terminus of the fluvial systems here may have been the northern lowlands. The NSM system does appear to be at least semicontiguous to the dichotomy and northern lowlands (Fassett & Head, 2008b; Irwin et al., 2005). Furthermore, Cusus Valles may have drained toward Indus Vallis, connecting it to the NSM system, and suggesting that this area was more regionally integrated than previous thought (Figure S6). We recognize that our study region does not extend all the way to the dichotomy; the northern part of the NSM system has been subsequently infilled by younger, geologically recent materials, thus obscuring the older stratigraphy. Future studies may shed further light on this problem.

In southwest Arabia Terra, the inverted channel systems show no clear connection to the valley networks south of Meridiani Planum, their possible source region (Figures 9 and S4). Water and sediment here were likely stored in floodplains and local paleolakes, consistent with the present low‐relief topography. This region may have been an ancient basin (or series of interconnected basins), which spanned for hundreds of kilometers. However, the current topography makes the present margins of the basins unclear. Elsewhere in Arabia Terra, completely preserved sediment routing pathways to the dichotomy are not observed, although this could be explained by regional denudation removing the evidence (e.g., northwest Arabia Terra; Fassett & Head, 2007; Hynek & Phillips, 2001; Moore, 1990). In summary, while some of the fluvial systems show possible pathways to the dichotomy, many do not. Regionally, sediment appears to have been stored in overbank deposits, local paleolakes, and regional‐scale basins, rather than being transported on to the dichotomy.

### 5.5 Chronology of the Fluvial Systems

To determine the relative chronology of the Arabia Terra fluvial systems, we analyzed their stratigraphic relationship to regional geologic units. On the global geologic map of Mars, the fluvial systems occur on regional geologic units that are middle to late Noachian in age (mNh; Tanaka et al., 2014; 3.9–3.7 Ga; Michael, 2013).

In southwest Arabia Terra, the fluvial systems almost always on the stratigraphically oldest exposed terrains, within erosional inliers. The fluvial systems here are superposed not only by up to ~1 km in vertical thickness of the etched units but also by numerous ~20‐ to 50‐km‐diameter and larger middle to late Noachian impact craters (Figures 9 and S4; Hynek & Di Achille, 2017). On the regional geologic map of Hynek and Di Achille (2017), the fluvial systems are mostly found overlying middle to late Noachian age geologic units (Nhc_1‐2_; Figure S4) and are clearly overlain by middle to late Noachian craters (C_2_) and by the middle Noachian to early Hesperian etched units (Nme_1‐3_ and HNme_u_). The formation age of the etched units in this region has been well constrained to between the late Noachian and Hesperian using their relationship to large impact craters (Hynek & Di Achille, 2017). Importantly, we find that the fluvial systems are always beneath the oldest etched unit (Nme_1_; middle to late Noachian; Hynek & Di Achille, 2017). Thus, in this region, the fluvial systems were active during the middle to late Noachian.

In central and eastern Arabia Terra, the fluvial systems are also overlain by the etched units, which in this region formed between the late Noachian and Noachian/Hesperian boundary (Fassett & Head, 2007). Throughout Arabia Terra, the reconstructed paleotopography of etched units (i.e., their preerosional surface) closely correlates to the distribution of identified inverted channel systems (Zabrusky et al., 2012; Figure S5), suggesting they were buried by the etched units. In addition, features indicative of fluvial systems are not observed within the etched units or other younger geologic units (Hynek & Di Achille, 2017; Tanaka et al., 2014).

The fluvial systems contained within the bedrock erosional valleys (in central and eastern Arabia Terra) are harder to establish an age, for, however, the initial formation age of the bedrock valley can provide a lower bound. These erosional valley networks are considered to have mostly formed around the Noachian/Hesperian boundary or early Hesperian (e.g., Naktong and Scamander Vallis; Bouley et al., 2009; Fassett & Head, 2008a; Hoke et al., 2011; Irwin et al., 2005). Naktong Vallis may have been locally reactivated in the early Hesperian, or possibly even the late Hesperian (e.g., Bouley et al., 2009; Howard et al., 2005). We note, however, that these reactivated regions predominantly occur in the upstream sections of Naktong Vallis, away from where the inverted systems are found and that this period of activity may have been more localized (Bouley et al., 2009). Given the thickness of the inverted fluvial deposits within the valleys (up to 100 m), it seems more likely that they were associated with the main phase of valley formation at the Noachian/Hesperian boundary or in the early Hesperian. Thus, the fluvial systems in the central and eastern Arabia Terra could have been active between the late Noachian and early Hesperian.

In summary, the relationship of the fluvial deposits to the regional stratigraphy suggests that fluvial activity in Arabia Terra occurred mostly between the mid‐Noachian and early Hesperian, although it is possible that some systems may have formed later or been reactivated with localized, late‐stage flows. The fluvial systems in southwest Arabia Terra may be older than those in the central and eastern regions, which are associated with erosional valley networks.

### 5.6 Consideration of Depositional Fluvial Systems for Early Martian Climate

Our observations in Arabia Terra add new geologic insights into the current conversation on the nature of early Mars paleoclimate. While the depositional fluvial systems we describe may be fragmented and discontinuous, their morphology and widespread regional occurrence nevertheless allow us to draw several conclusions about the environment in which they formed. Firstly, the relations of the fluvial systems to regional geologic units brackets their formation to mostly between the mid‐Noachian and early Hesperian. These thus represent some of the oldest fluvial deposits exposed in Mars's stratigraphic record. Fluvial systems have continuous lengths up to 100–200 km, though potentially ~10^3^ km long given the fragmented nature of preservation, indicating that water and sediment fluxes were sufficient to build depositional systems with long transport lengths. These are not local source to sink systems.

Moreover, many of the fluvial systems radiate away from local topographic highs and originate at multiple elevations (e.g., Figures 4, 5, and 9), which is more consistent with formation due to a distributed source of water (e.g., precipitation and runoff; Craddock & Howard, 2002) than the melt of regional ice sheets (e.g., Wordsworth et al., 2015). The origin of many of the fluvial systems occurs significant distances away from where ice sheets are predicted to form (Wordsworth et al., 2015). The absence of associated glacial landforms in Arabia Terra also argues against an “icy highlands” origin for the fluvial deposits.

Fluvial erosion incised 100‐ to 250‐m‐deep valleys into bedrock, generating sufficient sediment to cause accumulation of between 50‐ and 100‐m thicknesses of fluvial deposits. We find no evidence to suggest that these systems were emplaced by catastrophic flow processes. The fluvial deposits likely comprise stacked channel deposits built through vertical aggradation of the fluvial depositional system, strongly suggesting accumulation over geologically significant time durations rather than a sustained single episode of runoff. Fluvial stratigraphy is notoriously incomplete at preserving time and sediment accumulation rates are highly variable (Badgley & Tauxe, 1990; McRae, 1990). Thus, the depositional record within the inverted channel systems likely represents a small fraction of the overall time that fluvial systems were active. Using a range of sediment accumulation rates as measured from terrestrial stratigraphic records of between 0.1 and 1.0 m/kyr (McRae, 1990; Miall, 2015) indicates that 50–100 m of fluvial deposits would take ~10^4^–10^6^ years to accumulate. Continuous deposition is also inconsistent with the development of lake chain systems that involved periodic overtopping events (Fassett & Head, 2008b).

Furthermore, a recent hydrological reconstruction of terrestrial inverted paleochannels in the Californian Mojave Desert has implications for interpreting the timescales and climatic conditions for Martian systems. Miller et al. (2018) mapped ridges (~1‐m relief) that comprise a distributary inverted paleochannel network of the Mojave River feeding southern Lake Coyote intermittently in the late Quaternary. At the distal end of this Minneola fluvial plain of the Mojave River, Miller et al. (2018) interpret multiple, intermittent lacustrine events and document at least five lake‐rise pulses through stratigraphic sections and age control from radiocarbon dating of shallow water lacustrine fossils. Importantly, this sequential development of inverted paleochannels, formed in multiple flows between ~25 and 15 ka within a narrow elevation range (<5 m), is not evident from planimetric pattern. Absent sedimentology data and accurate dates, the radiating ridge pattern might be erroneously interpreted from orbital data alone as a single event fluvial deposit. The Lake Coyote analog underpins the possibility that Martian inverted channels may similarly develop from multiphase depositional events over a long timescale with potentially variable climatic conditions (wet and dry).

This evidence points to a mid‐Noachian to early Hesperian surface environment in Arabia Terra that was potentially characterized by prolonged episodes of surface aqueous activity enabling the extensive downstream elongation of depositional river systems, commonly across regional‐scale basins. Thus, it would appear from this evidence that the regional early Mars climate was able to support persistent precipitation and runoff, which mirrors similar interpretations based on the analysis of valley networks (e.g., Craddock & Howard, 2002; Hoke & Hynek, 2009; Hynek et al., 2010; Matsubara et al., 2011), possibly in an arid to semiarid setting (e.g., Ramirez & Craddock, 2018). Exploring the early Mars paleoclimate conundrum requires detailed geologic analyses of aqueously transported sedimentary deposits that are placed within a reasonable stratigraphic framework to a degree constrainable from available orbital observations.

## 6 Conclusions

The Arabia Terra region of Mars records a series of regionally extensive depositional fluvial sediment routing systems. These comprise exhumed fluvial channel deposits that are expressed as inverted topography that record at least 50‐ to 100‐m‐thick stratigraphy. Associated with these are possible floodplain deposits, paleolake basins and inverted paleolake deposits, and partially filled valleys and basins. The inverted channel systems and surrounding deposits probably comprise indurated sandstone and conglomerate channel‐belt sedimentary bodies that are inset within finer‐grained, mud‐rich overbank deposits.

Some of the inverted channel systems are found as isolated systems, usually in low‐lying plains, and many systems are found in proximity to regionally sourced erosional valley networks and paleolake basins (Fassett & Head, 2008b; Hynek et al., 2010). These commonly record downslope transitions: Inverted channel deposits occur in the downslope parts of valley networks and emerge from valley networks to form unconfined inverted channel systems. These transitions could simply be due to topography, or possibly changes in water or sediment supply, or base‐level fluctuations. Fluvial downcutting must have occurred initially to form the erosional valleys and basins before they were partially filled following base‐level rise.

The fluvial systems show catchments with both regional and local extents, although in several cases no clear catchment is preserved at all. In central and eastern Arabia Terra, fluvial systems may have transported sediment toward the planetary dichotomy and northern lowlands. However, this is not clearly the case elsewhere in Arabia Terra, where much of the sediment appears to have been stored in local paleolakes and regional‐scale basins. The fluvial systems in Arabia Terra most likely developed over a protracted period between the mid‐Noachian and early Hesperian, and their formation was likely intermittent. Additional fluvial systems may be buried beneath the surface; the present surface exposure may only represent part of the overall preserved fluvial stratigraphy. Finally, the fluvial systems are consistent with forming under an early Mars climate that supported prolonged and persistent precipitation and runoff.

## Supporting information

Supporting Information S1
