# Supplementary material for: A Diverse Array of Fluvial Depositional Systems in Arabia Terra: Evidence for mid‐Noachian to Early Hesperian Rivers on Mars
Source: J Geophys Res Planets. 2019 Jul 22;124(7):1913–34. doi: 10.1029/2019JE005976 (PMC6774298; doi:10.1029/2019JE005976)
Supplement: Supplementary file 1 — Supporting Information S1 [file JGRE-124-1913-s001.pdf]

AGU PUBLICATIONS

*Journal of Geophysical Research Planets*

Supporting Information for

# A Diverse Array of Fluvial Depositional Systems in Arabia Terra: Evidence for mid-Noachian to Early Hesperian Rivers on Mars

Joel M. Davis[1], Sanjeev Gupta[2], Matthew Balme[3], Peter M. Grindrod[1], Peter Fawdon[3], Zachary I. Dickeson[1], Rebecca M.E. Williams[4]

[1]Department of Earth Sciences, Natural History Museum, Cromwell Road, Kensington, London, SW7 5BD, UK

[2]Department of Earth Sciences and Engineering, Imperial College London, London, SW7 2AZ, UK

[3]School of Physical Sciences, The Open University, Milton Keynes, Buckinghamshire, MK7 7EA, UK

[4]Planetary Science Institute, 1700 E. Fort Lowell, Suite 106, Tucson, Arizona 85719, USA

## Contents of this file

Figures S1 to S6
Tables S1 to S4

## Introduction

This file contains further examples and topographic profiles of the inverted channel deposits, as well as a table detailing the characteristics of the largest systems. Similarly, there are also further examples of the paleolakes found in association with the inverted channel deposits and a table showing the locations of all the identified systems. There are also figures showing how the distribution of inverted channels compares to (1) the reconstructed paleo-topography in Arabia Terra and (2) regionally-mapped geological units. We also show the potential pathways from the terminus of Cusus Valles to Indus Vallis. Finally, we include tables showing the different datasets used in the study (e.g., HiRISE and CTX stereo pairs, image numbers).

| Instrument | Image 1 | Image 2 | Center lat. | Center long. | DEM spatial resolution (m/pixel) |
|---|---|---|---|---|---|
| HiRISE | ESP_038428_1880 | ESP_047461_1810 | 7.82°N | 11.25°W | 1 |
| HiRISE | ESP_041949_1880 | ESP_043162_1880† | 8.1°N | 10.88°W | 1 |
| HiRISE | ESP_036898_1895 | ESP_036832_1895 | 9.15°N | 5.48°W | 1 |
| HiRISE | ESP_012648_1815 | ESP_012714_1815 | 1.38°N | 7.68°E | 1 |
| CTX | P18_008139_1900_XN_10N004W | P22_009576_1903_XN_10N004W | 10.09°N | 4.26°W | 20 |
| CTX | F23_044731_1859_XI_05N001W | J04_046287_1859_XI_05N001W | 5.94°N | 1.11°W | 20 |
| CTX | D06_029487_1841_XI_04N349W | D06_029421_1841_XI_04N349W | 4.09°N | 10.38°E | 20 |
| CTX | B17_016458_1864_XI_06N329W | P04_002758_1880_XI_08N329W | 8.1°N | 30.4°E | 20 |
| CTX | P16_007228_1973_XI_17N329W | P15_007017_1969_XI_16N329W | 16.95°N | 30.5°E | 20 |
| CTX | G12_022945_1914_XI_11N312W | G13_023235_1914_XI_11N312W | 11.42°N | 47.24°E | 20 |
| CTX | B10_013543_1964_XI_16N310W | B11_013820_1965_XI_16N310W | 16.51°N | 49.75°E | 20 |

**Table S1.** HiRISE and CTX resolution DEMs produced of the Arabia Terra study region.

†This DEM was not produced from a true HiRISE stereo pair.

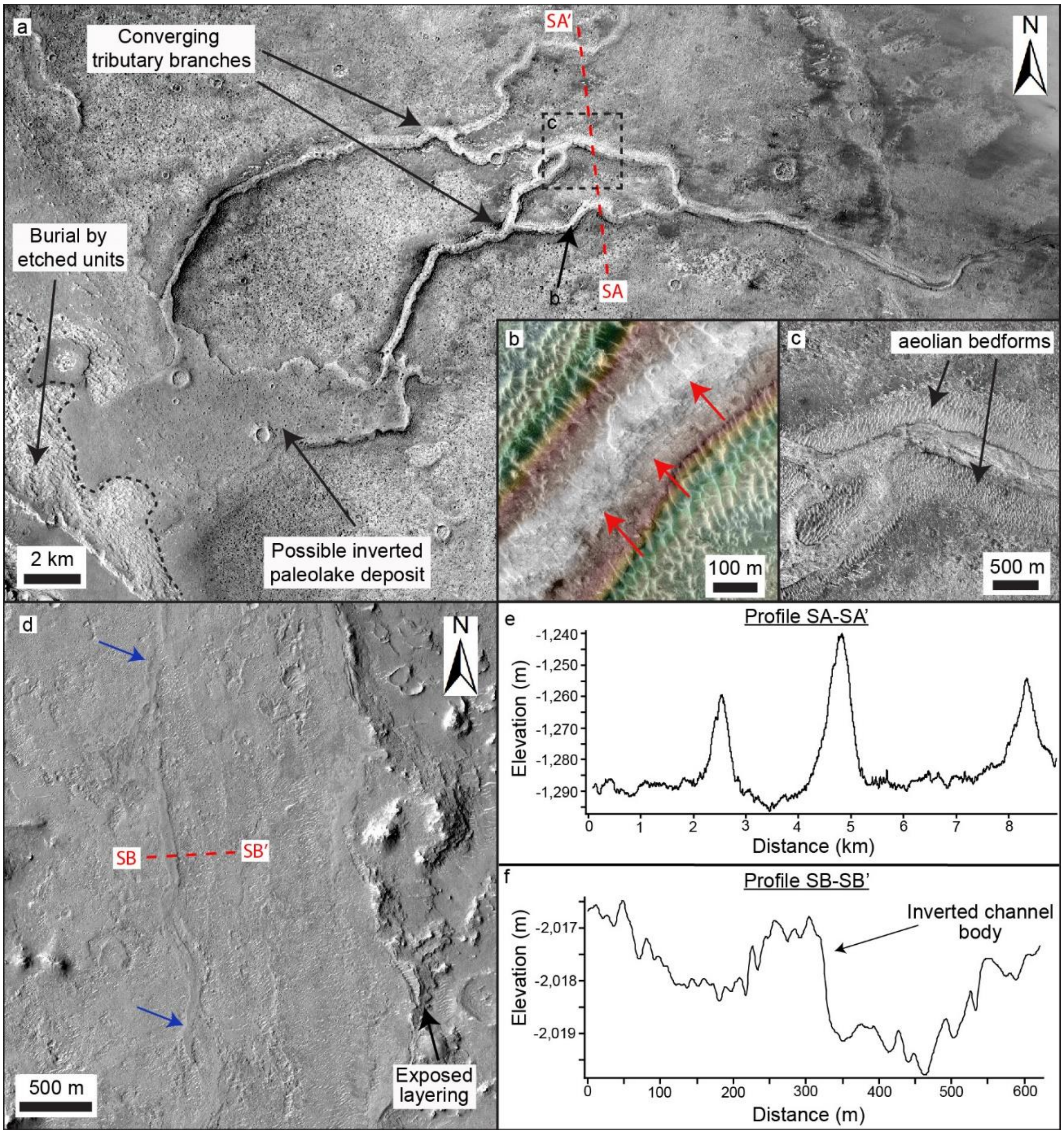


**Figure S1**. Examples of inverted channels in Arabia Terra with different preservation styles. (a) CTX mosaic of branching and anabranching inverted channel in south Arabia Terra, named Meridiani Serpentes, which terminates in possible paleolake deposit and has been buried by the etched units. This inverted channel is up to ~ 1 km wide and ~ 50 m high as shown in the profile extracted from a HiRISE DEM in (e). (b) HiRISE DEM overlaid on HiRISE image showing sub-horizontal layering in channel margins (red arrow). (c) HiRISE image showing aeolian bedforms which are concentrated around the margins of the inverted channel, consistent with it comprising a sandstone body. (d) HiRISE image of an inverted channel (highlighted by blue arrows), with little topographic expression. The inverted channel here is ~ 100 m wide, but only ~ 1 m high, as shown by the profile extracted from a HiRISE DEM in (f).

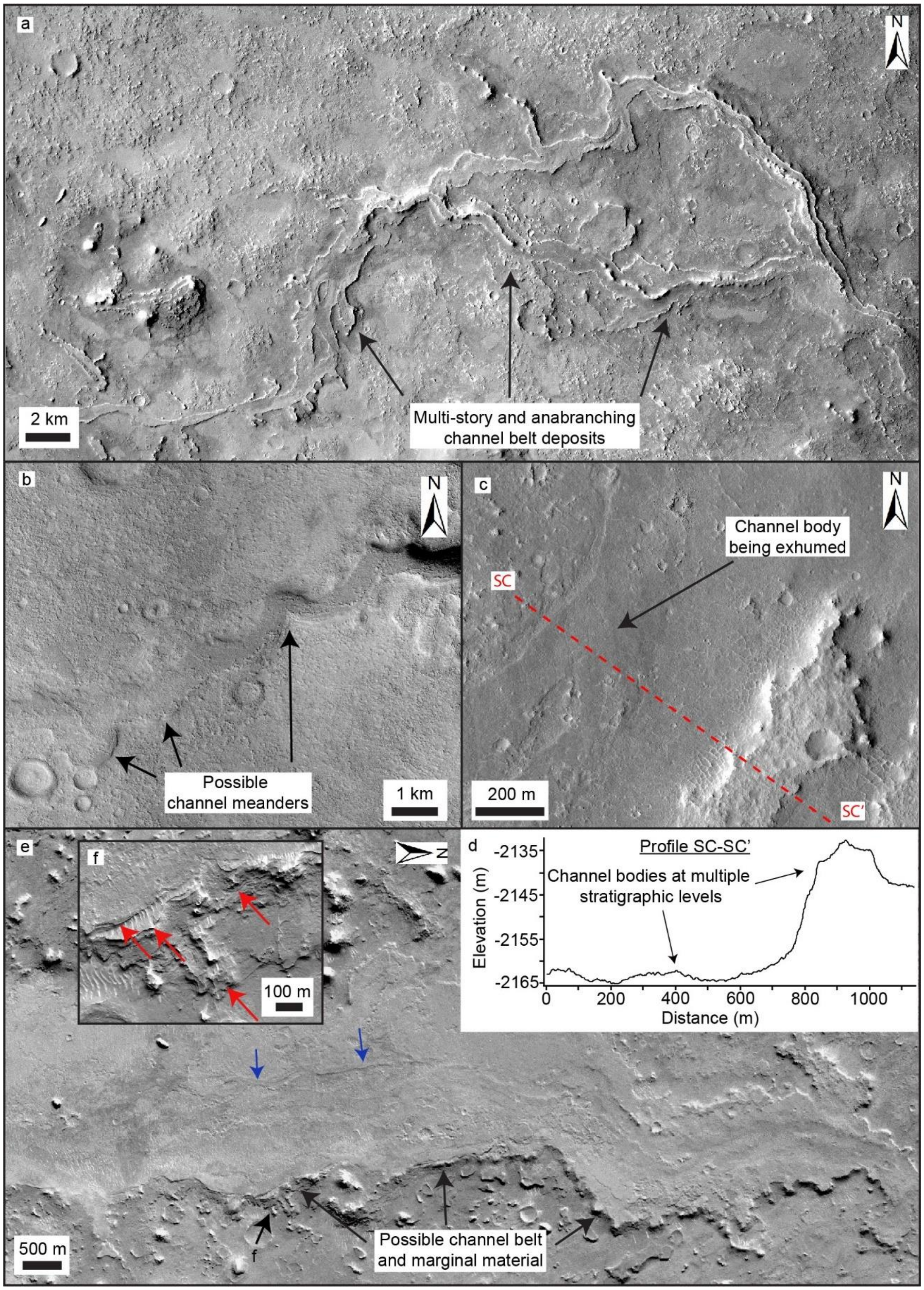


**Figure S2:** Examples of inverted channels in Arabia Terra with different planview morphologies. (a) CTX image of branching and anabranching inverted channel deposits.

Multiple channels have amalgamated laterally and aggraded vertically (forming a multi-story profile), suggesting this is a channel belt deposit. (b) CTX mosaic showing possible meanders in an inverted channel within the Phison Patera basin. (c) HiRISE image showing inverted channels at different stratigraphic levels, consistent with a channel belt deposit that has aggraded. The channel at the left of the image has only been partially exhumed. (d) Topographic profile extracted from a HiRISE DEM of above inverted channels. (e) CTX image showing channel belt and associated marginal (overbank) material deposit. (f) HiRISE image showing layering exposed in the marginal material in (e).

| Lat. | Long. | Longest contiguous segment (km) | Max. width (m) | Max ridge height (m) | DTM used | Bound by erosional valley? | Contiguous with erosional valley? | Observations |
|---|---|---|---|---|---|---|---|---|
| 16 °N | 49.7 °E | 48.3 | ~ 1100 | 50-70 | CTX | Yes, initially | Yes | Branching, sinuous, anabranching. (See Figure 5e). |
| 7.8 °N | 11.4°W | 84.8 | ~ 1200 | ~ 25 | HiRISE | No | No | Branching, sinuous, anabranching. (See Figure 2a). |
| 7.4 °N | 7.2 °W | 67.4 | ~ 700 | ~ 50 | MOLA | No | No | Branching, sinuous, anabranching. (See Figure 9b). |
| 7.5 °N | 30.6 °E | 114 | ~ 450 | ~ 70 | CTX | Yes | Yes | Branching, anabraching. Terraces present in valley. (See Figure 4b). |
| 17.5 °N | 30.0 °E | 132 | ~ 1300 | ~ 50 | CTX | Yes | Yes | Branching, sinuous, anabranching. (See Figure 4e) |
| 29.2 °N | 61.0 °E | 92.8 | ~ 2600 | ~ 100 | HRSC | No | Yes | Sinuous, anabranching. Incised by Auqakah Vallis. (See Figure 8b). |
| 19.3 °N | 37.1 °E | 23.9 | ~ 600 | - | | Yes | Yes | Linear, sinuous. Terraces present in valley. |
| 4.4 °N | 10.3 °E | 107.2 | ~ 1000 | ~ 80 | CTX | No | Yes | Branching. |
| 1.2 °N | 7.5 °E | 128.6 | ~ 700 | ~ 50 | HiRISE | No | Yes | Branching, anabranching. (See Figure S1a). |
| 9.3 °N | 5.2 °W | 102.6 | ~ 500 | ~ 50 | HiRISE | Yes | Yes | Sinuous, branching. (See Figure S2e) |
| 9.5 °N | 4.3 °W | 105.1 | ~ 550 | ~ 70 | CTX | No | No | Sinuous, branching. |
| 10.3 °N | 7.5 °W | 192.4 | ~ 900 | ~ 50 | MOLA | No | No | Branching, sinuous, anabranching. |
| 6.2 °N | 2.5 °E | 126 | ~ 500 | ~ 20 | MOLA | No | No | Linear. |
| 4.3 °N | 0.3 °W | 94.9 | ~ 200 | ~ 20 | CTX | No | No | Branching, low junction angles |
| 11.2 °N | 4.2 °E | 66.4 | ~ 900 | ~ 10 | MOLA | No | No | Branching, sinuous, anabranching. (See Figure S2a). |
| 19.4 °N | 37.6 °E | 43.1 | ~ 1300 | ~ 50 | CTX | Yes, initially | Yes | Branching, sinuous, anabranching. (See Figure 5d). |

**Table S2:** Table showing characteristics for the longest inverted channel segments in Arabia Terra.

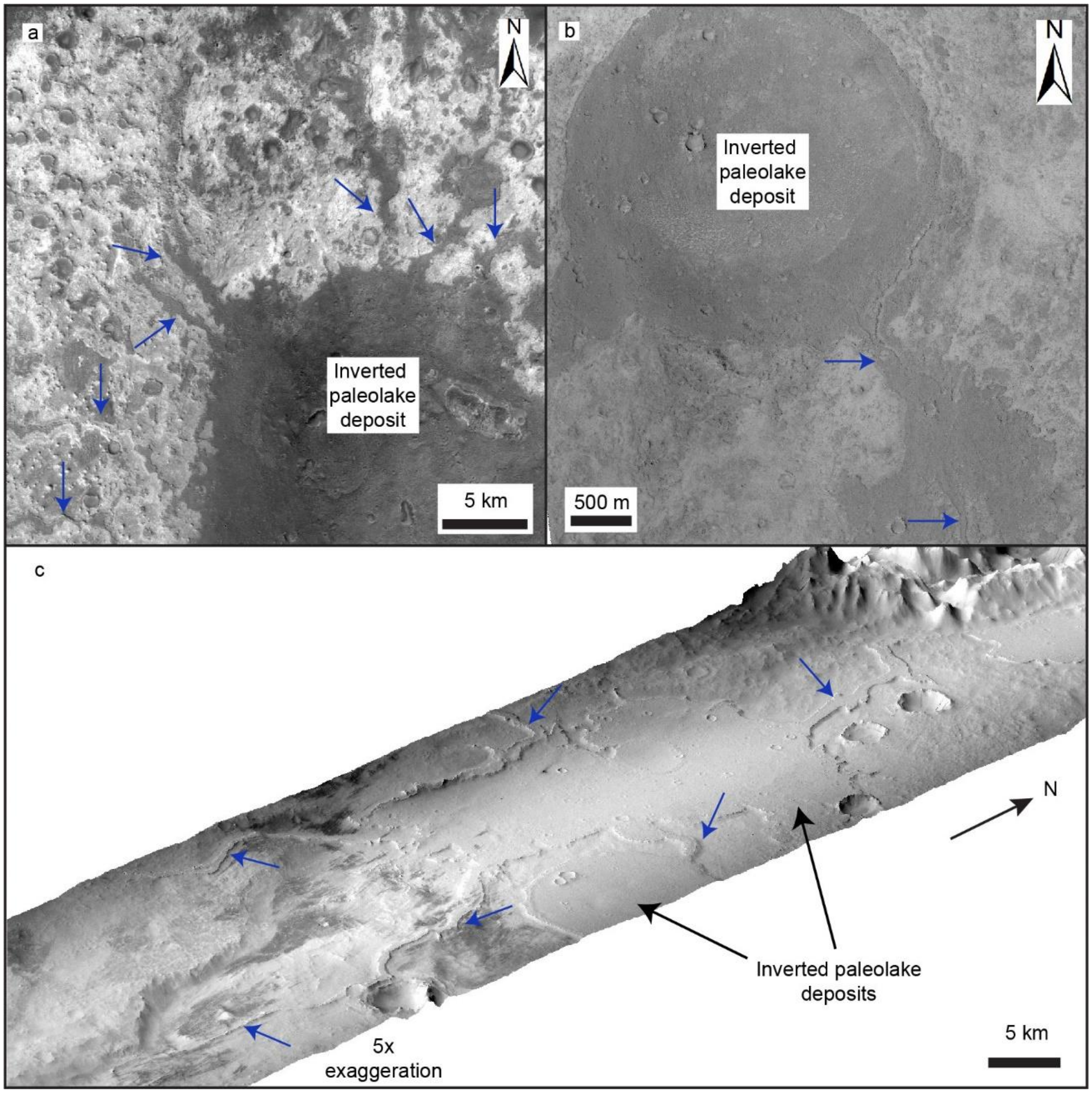


**Figure S3:** Examples of paleolakes associated with the inverted channels (blue arrows) in Arabia Terra. (a) CTX mosaic of inverted paleolake deposit near Mawrth Vallis region. (b) HiRISE image of inverted paleolake deposit in southwest Arabia Terra. (c) Perspective CTX view of inverted channels and associated paleolake deposits at the end of Locras Valles.

| Latitude | Longitude | Open or Closed? | Morphology |
|---|---|---|---|
| 19.58°N | 16.99°W | Closed | Inverted paleolake deposit |
| 19.89°N | 16.92°W | Open | Inverted paleolake deposit |
| 19.88°N | 16.71°W | Open | Inverted paleolake deposit |
| 6.27°N | 11.62°W | Closed | Inverted paleolake deposit |
| 7.04°N | 11.07°W | Unclear | Inverted paleolake deposit |
| 7.16°N | 11.02°W | Open | Inverted paleolake deposit |
| 8.15°N | 10.99°W | Open | Basin containing sediment deposits |
| 7.83°N | 8.48°W | Open | Inverted paleolake deposit |
| 6.41°N | 8.45°W | Open | Inverted paleolake deposit |
| 6.37°N | 8.30°W | Open | Inverted paleolake deposit |
| 9.99°N | 7.74°W | Open | Inverted paleolake deposit |
| 5.51°N | 7.62°W | Open | Inverted paleolake deposit |
| 9.96°N | 7.51°W | Open | Inverted paleolake deposit |
| 5.82°N | 3.35°E | Open | Basin with terraces |
| 11.18°N | 4.73°E | Unclear | Inverted paleolake deposit |
| 1.22°N | 7.44°E | Unclear | Inverted paleolake deposit |
| 13.61°N | 8.48°E | Open | Inverted paleolake deposit |
| 16.18°N | 15.23°E | Open | Inverted paleolake deposit |
| 21.06°N | 19.89°E | Open | Inverted paleolake deposit |
| 23.88°N | 19.94°E | Closed | Basin containing sediment deposits |
| 21.75°N | 20.42°E | Closed | Inverted paleolake deposit |
| 20.97°N | 20.48°E | Close | Inverted paleolake deposit |
| 23.51°N | 20.56°E | Closed | Basin containing sediment deposits |
| 25.58°N | 24.97°E | Open | Inverted paleolake deposit |
| 24.90°N | 25.12°E | Open | Inverted paleolake deposit |
| 25.24°N | 25.86°E | Closed | Basin containing sediment deposits |
| 6.57°N | 26.08°E | Closed | Inverted paleolake deposit |
| 4.92°N | 26.29°E | Closed | Inverted paleolake deposit |
| 9.97°N | 26.33°E | Closed | Inverted paleolake deposit |
| 5.42°N | 26.83°E | Closed | Basin containing sediment deposits |
| 24.12°N | 34.19°E | Closed | Basin containing sediment deposits |
| 17.83°N | 34.45°E | Open | Inverted paleolake deposit |
| 26.79°N | 36.55°E | Open | Basin containing sediment deposits |
| 16.30°N | 39.42°E | Closed | Inverted paleolake deposit |
| 13.14°N | 46.84°E | Open | Inverted paleolake deposit |
| 11.01°N | 47.31°E | Open | Inverted paleolake deposit |
| 11.62°N | 47.33°E | Closed | Inverted paleolake deposit |
| 10.78°N | 47.53°E | Closed | Inverted paleolake deposit |
| 18.46°N | 47.68°E | Open | Inverted paleolake deposit |
| 11.33°N | 47.76°E | Closed | Inverted paleolake deposit |
| 17.54°N | 48.25 | Closed | Inverted paleolake deposit |
| 25.56°N | 48.51 | Closed | Inverted paleolake deposit |

| | | | |
|---|---|---|---|
| 16.50°N | 49.51 | Closed | Inverted paleolake deposit |
| 18.77°N | 50.28 | Closed | Inverted paleolake deposit |
| 26.23°N | 51.51 | Open | Basin |
| 22.22°N | 53.11 | Closed | Inverted paleolake deposit |
| 29.68°N | 66.65 | Open | Inverted paleolake deposit |
| 32.26°N | 67.30 | Closed | Basin containing sediment deposits |

**Table S3:** Table showing characteristics of the paleolake basins and deposits identified in association with the inverted channels in Arabia Terra.

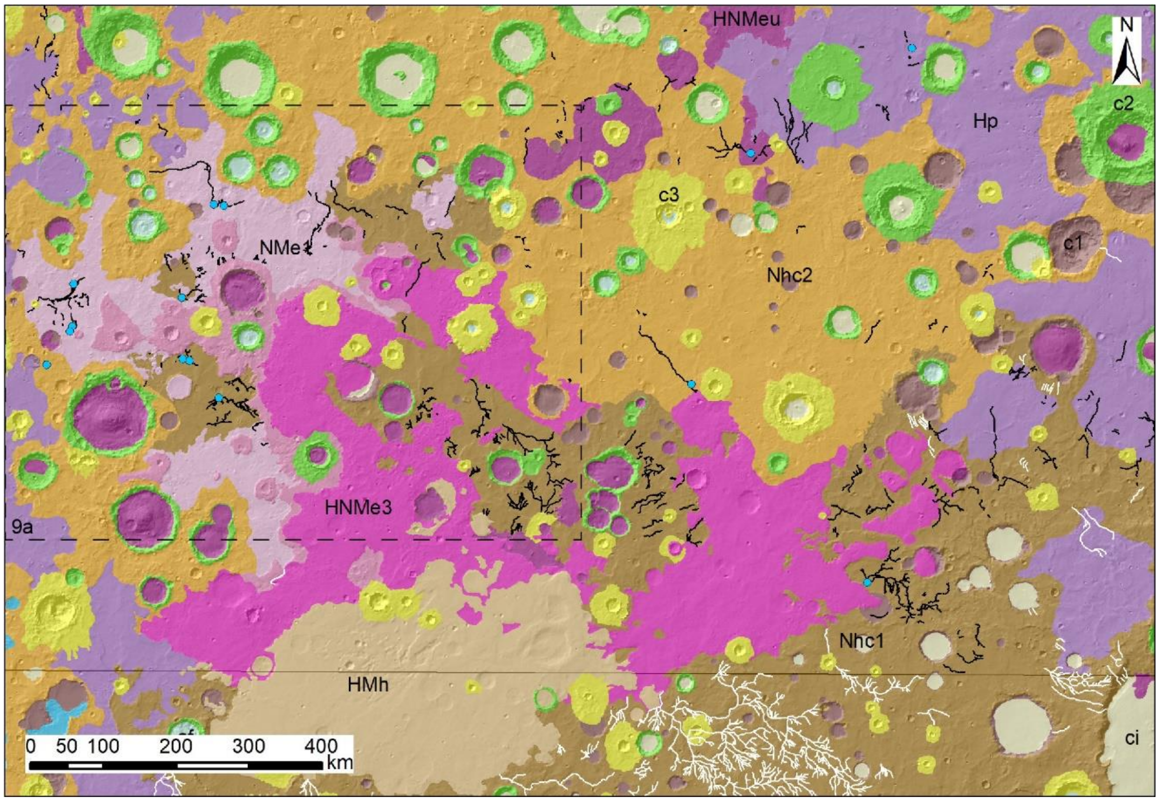


**Figure S4:** Geological units mapped by Hynek and Di Achille (2017) overlaid on a shaded relief map of MOLA topography and their relationship to the inverted channels (black lines). Most inverted channels occur on two geological units: $Nhc_1$ and $Nhc_2$, which range from mid to late Noachian in age. These units are at the base of the exposed stratigraphy of Meridiani Planum. $Nhc_1$ and $Nhc_2$ are overlain by the etched units: $Nme_{1-3}$ and $HNme_u$, which are considered mid Noachian to early Hesperian. This constrains the likely formation period of the inverted channels in this region to the mid to late Noachian. Although some inverted channels occur on the lowermost etched unit $NMe_1$ as mapped from 100 m/pixel data (e.g., northwest region of figure), when viewed at a higher resolution, the inverted channels are being exhumed from beneath the etched units (e.g., Figure 2a). More broadly

throughout Arabia Terra, the inverted channels are found mostly on the global geological unit mNh, which is middle Noachian in age (Tanaka et al., 2014). Geological units adapted from Hynek and Di Achille (2017). The GIS data for the Hynek and Di Achille (2017) map can be downloaded here: https://pubs.er.usgs.gov/publication/sim3356.

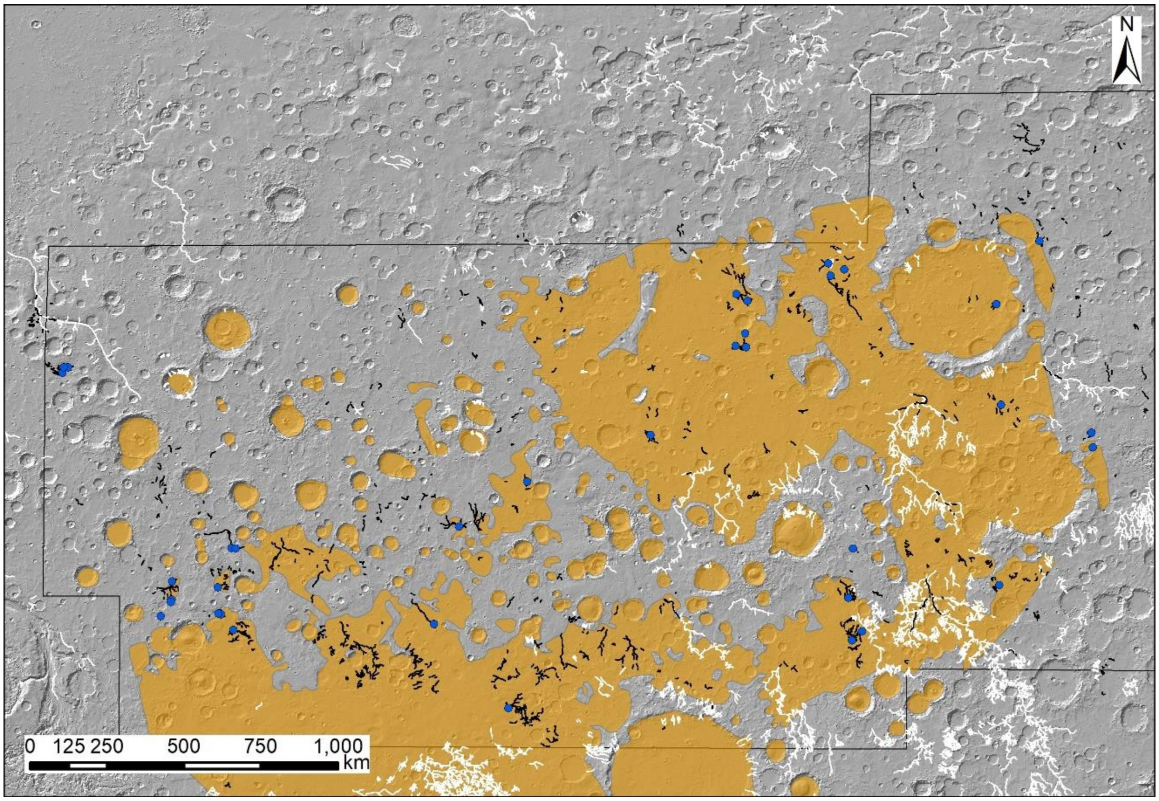


**Figure S5:** Reconstructed paleo-surface (adapted from Zabrusky et al., 2012) showing the former extent of the etched units in the western Arabia Terra region, overlaid on a shaded relief map of MOLA topography. ~ 80% of the mapped inverted channels (black lines) are spatially associated with this surface, suggesting that the etched units may have protected the inverted channels from erosion. In those areas where the etched units did not extend to (e.g., northwest Arabia Terra), the inverted channels may have been eroded away.

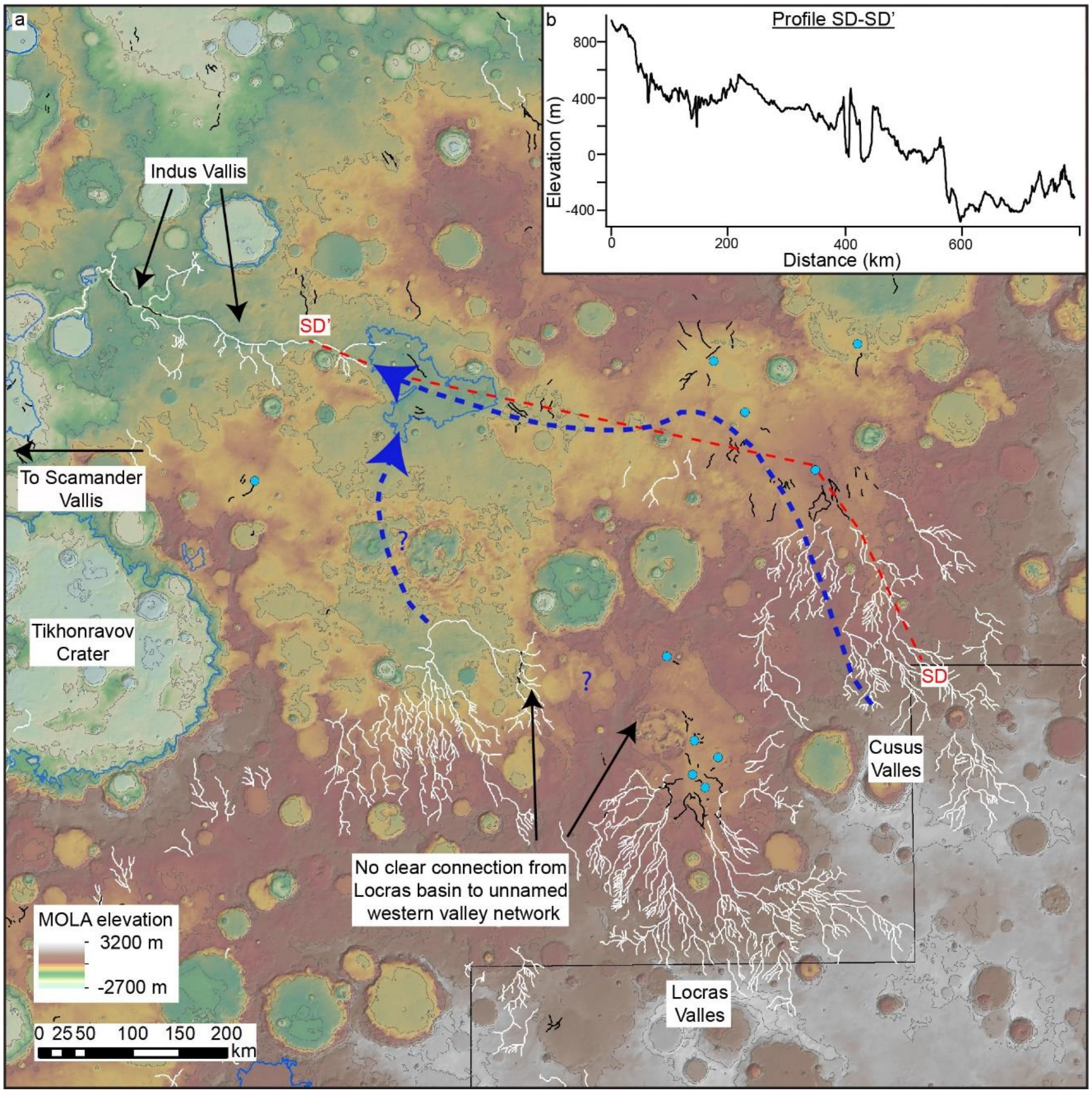


**Figure S6**. (a) MOLA topography overlaid on shaded relief map (contours shown at 500 m intervals) showing potential pathways between Cusus and Locras Valles and Indus Vallis. Cusus Valles may have drained into a large, open basin which ultimately connected to Indus Vallis and thus the Naktong, Scamander, and Mamers Vallis system. The basin at the end of Locras Valles does not have a clear outlet, suggesting it was not connected to the NSM system. However, the unnamed valley network to the west of Locras Valles may have drained towards Indus Vallis, although the connection has been buried by subsequent cratering. The addition of these two valley networks to the NSM system would significantly expand the catchment of the system, which is already the equivalent to continental drainage basins. (b) Profile extracted from MOLA DEM showing decrease in elevation from Cusus Valles towards Indus Vallis.

| Figure | Instrument | Image ID |
|---|---|---|
| 1a | MOLA | |
| 2a | CTX | B03_010829_1892_XI_09N012W; |
| | | B01_009840_1885_XN_08N010W; |
| | | D09_030714_1876_XI_07N011W; |
| | | G23_027180_1889_XI_08N011W |
| 2b | HiRISE | ESP_038428_1880 |
| 2c | HiRISE DEM | ESP_038428_1880, |
| | | ESP_047461_1810 |
| 2d | HiRISE, CTX DEM | ESP_053236_1905, |
| | | P22_009576_1903_XN_10N004W, |
| | | P18_008139_1900_XN_10N004W |
| 2e | HiRISE | PSP_004091_1845 |
| 2f | HiRISE | ESP_044085_1900 |
| 2g | HiRISE | ESP_028881_1855 |
| 3a | HiRISE | ESP_041949_1880 |
| 3b | HiRISE, CTX | ESP_033586_2065, |
| | | D16_033296_2064_XN_26N308W |
| 3c | CTX | D04_028881_1843_XI_04N007W |
| 3d | CTX | B03_010880_2023_XN_22N326W, |
| | | P14_006529_2030_XN_23N325W |
| 3e | HiRISE | ESP_049597_1875 |
| 3f | CTX | P17_007730_1855_XI_05N356W |
| 4a | MOLA | |
| 4b | CTX | J06_047288_1870_XI_07N329W, |
| | | P04_002758_1880_XI_08N329W, |
| | | P19_008375_1874_XN_07N329W |
| 4c | CTX DEM (profile) | B17_016458_1864_XI_06N329W, |
| | | P04_002758_1880_XI_08N329W |
| 4d | CTX DEM (profile) | B17_016458_1864_XI_06N329W, |
| | | P04_002758_1880_XI_08N329W |
| 4e | CTX | P13_006160_1977_XN_17N330W, |
| | | P15_006872_1983_XI_18N329W |
| 4f | CTX | P04_002758_1880_XI_08N329W, |
| | | P19_008375_1874_XN_07N329W |
| 4g | CTX | P04_002758_1880_XI_08N329W, |
| | | P19_008375_1874_XN_07N329W |
| 4h | HiRISE, CTX | PSP_006872_1985, |
| | | P15_006872_1983_XI_18N329W |
| 5a | MOLA | |
| 5b | MOLA | |
| 5c | MOLA | |

| | | |
|---|---|---|
| 5d | CTX | G12_022945_1914_XI_11N312W, P17_007557_1901_XI_10N313W, D17_033771_1915_XI_11N313W, D04_028668_1909_XI_10N312W |
| 5e | CTX | B19_017143_1957_XN_15N311W, P02_001821_1958_XN_15N309W, P03_002032_1959_XN_15N310W, P04_002599_1958_XN_15N310W, P07_003588_1958_XN_15N310W, P07_003799_1961_XN_16N311W |
| 5f | CTX | P17_007557_1901_XI_10N313W, D17_033771_1915_XI_11N313W |
| 5g | CTX | D04_028668_1909_XI_10N312W, G12_022945_1914_XI_11N312W |
| 5h | CTX | P07_003588_1958_XN_15N310W, P03_002032_1959_XN_15N310W |
| 6a | MOLA, THEMIS-IR Day | |
| 6b | CTX | B21_017776_2087_XN_28N315W, F04_037463_2089_XN_28N314W, G21_026558_2096_XN_29N315W, P07_003654_2082_XN_28N314W, P08_004221_2082_XN_28N313W |
| 6c | CTX | G18_025160_2070_XN_27N308W, P05_002889_2089_XN_28N309W, P05_003100_2079_XN_27N309W |
| 7a | MOLA, THEMIS-IR Day | |
| 7b | CTX | B17_016445_1890_XN_09N334W, B18_016656_1880_XN_08N334W, D01_027627_1876_XI_07N333W, B19_017012_1877_XN_07N333W |
| 7c | CTX | B17_016445_1890_XN_09N334W |
| 7d | CTX | B18_016656_1880_XN_08N334W, B19_017012_1877_XN_07N333W, D01_027627_1876_XI_07N333W |
| 7e | CTX | B18_016656_1880_XN_08N334W, B19_017012_1877_XN_07N333W |
| 8a | MOLA, THEMIS-IR Day | |
| 8b | CTX | J14_050214_2098_XN_29N299W, P04_002559_2098_XN_29N299W, P12_005895_2100_XI_30N299W, |
| 8c | HRSC DEM (profile) | h7357_0000 |

| | | |
|---|---|---|
| 8d | CTX | J14_050214_2098_XN_29N299W, P04_002559_2098_XN_29N299W, |
| 8e | CTX | J14_050214_2098_XN_29N299W |
| 9a | MOLA | |
| 9b | CTX | D04_028881_1843_XI_04N007W |
| 9c | MOLA (profile) | |
| | | |
| S1a | CTX | D06_029698_1813_XI_01N352W, G23_027298_1822_XI_02N352W, P04_002627_1808_XN_00N352W |
| S1b | HiRISE, HiRISE DEM | ESP_012648_1815, ESP_012714_1815 |
| S1c | HiRISE | ESP_012648_1815 |
| S1d | HiRISE | ESP_036898_1895 |
| S1e | HiRISE DEM (profile) | ESP_012648_1815, ESP_012648_1815 |
| S1f | HiRISE DEM (profile) | ESP_036898_1895, ESP_036832_1895 |
| S2a | CTX | B01_010090_1923_XI_12N355W |
| S2b | CTX | P08_004221_2082_XN_28N313W, P07_003654_2082_XN_28N314W |
| S2c | HiRISE | ESP_041949_1880 |
| S2d | HiRISE DEM (profile) | ESP_041949_1880, ESP_043162_1880 |
| S2e | CTX | F03_036832_1891_XI_09N005W |
| S2f | HiRISE | ESP_036898_1895 |
| S3a | CTX | D20_034960_2000_XN_20N017W, G11_022367_1996_XN_19N017W |
| S3b | HiRISE | ESP_057298_1865 |
| S3c | CTX DEM (perspective view) | G12_022945_1914_XI_11N312W, G12_022945_1914_XI_11N312W |
| S4 | MOLA hillshade | |
| S5 | MOLA hillshade | |
| S6a | MOLA | |
| S6b | MOLA DEM (profile) | |

**Table S4.** Figure instrument and image ID numbers.

**References**


Hynek, B.M., & Di Achille, G. (2017). Geological map of the Meridiani Planum, Mars. USGS Scientific Investigations Series (Map 3356, scale 1:2,000,000). https://doi.org/10.3133/sim3356

Williams, R.M.E., Irwin, R.P., Zimbelman, J.R., Chidsey, T.C., & Eby, D.E. (2011). Field guide to exhumed paleochannels near Green River, Utah: Terrestrial analogs for sinuous ridges on Mars. Geological Society of America Special Publication, 483, 483–505. https://doi.org/10.1130/2011.2483(29).

Zabrusky, K., Andrews-Hanna, J.C., & Wiseman, S.M. (2012). Reconstructing the distribution and depositional history of the sedimentary deposits of Arabia Terra, Mars. Icarus, 220, 311–330. https://doi.org/10.1016/j.icarus.2012.05.007.
